# Genomic diversity and biogeographic distributions of a novel lineage of bacteriophages that infect marine OM43 bacteria

**DOI:** 10.1128/spectrum.04942-22

**Published:** 2023-08-21

**Authors:** Mingyu Yang, Sen Du, Zefeng Zhang, Qian Xia, He Liu, Fang Qin, Zuqing Wu, Hanqi Ying, Yin Wu, Jiabing Shao, Yanlin Zhao

**Affiliations:** 1 Fujian Provincial Key Laboratory of Agroecological Processing and Safety Monitoring, College of Life Sciences, Fujian Agriculture and Forestry University, Fuzhou, China; 2 Key Laboratory of Marine Biotechnology of Fujian Province, Institute of Oceanology, Fujian Agriculture and Forestry University, Fuzhou, China; Swansea University, Swansea, United Kingdom

**Keywords:** OM43 clade, OM43 phages, comparative genomics, novel phage groups

## Abstract

The marine methylotrophic OM43 clade is considered an important bacterial group in coastal microbial communities. OM43 bacteria, which are closely related to phytoplankton blooms, have small cell sizes and streamlined genomes. Bacteriophages profoundly shape the evolutionary trajectories, population dynamics, and physiology of microbes. The prevalence and diversity of several phages that infect OM43 bacteria have been reported. In this study, we isolated and sequenced two novel OM43 phages, MEP401 and MEP402. These phages share 90% of their open reading frames (ORFs) and are distinct from other known phage isolates. Furthermore, a total of 99 metagenomic viral genomes (MVGs) closely related to MEP401 and MEP402 were identified. Phylogenomic analyses suggest that MEP401, MEP402, and these identified MVGs belong to a novel subfamily in the family *Zobellviridae* and that they can be separated into two groups. Group I MVGs show conserved whole-genome synteny with MEP401, while group II MVGs possess the MEP401-type DNA replication module and a distinct type of morphogenesis and packaging module, suggesting that genomic recombination occurred between phages. Most members in these two groups were predicted to infect OM43 bacteria. Metagenomic read-mapping analysis revealed that the phages in these two groups are globally ubiquitous and display distinct biogeographic distributions, with some phages being predominant in cold regions, some exclusively detected in estuarine stations, and others displaying wider distributions. This study expands our knowledge of the diversity and ecology of a novel phage lineage that infects OM43 bacteria by describing their genomic diversity and global distribution patterns.

**IMPORTANCE:**

OM43 phages that infect marine OM43 bacteria are important for host mortality, community structure, and physiological functions. In this study, two OM43 phages were isolated and characterized. Metagenomic viral genome (MVG) retrieval using these two OM43 phages as baits led to the identification of two phage groups of a new subfamily in the family *Zobellviridae*. We found that group I MVGs share similar genomic content and arrangement with MEP401 and MEP402, whereas group II MVGs only possess the MEP401-type DNA replication module. Metagenomic mapping analysis suggests that members in these two groups are globally ubiquitous with distinct distribution patterns. This study provides important insights into the genomic diversity and biogeography of the OM43 phages in the global ocean.

## INTRODUCTION

Viruses are widely recognized as the most abundant biological entities in the ocean and play pivotal roles in the marine microbial loop and biogeochemical cycles. It has been estimated that viral shunts produce about 10 billion tons of carbon per day by releasing organic matter, which is considered fundamental to nutrient cycling, promoting productivity of the oceans (1 – 3). As the major top-down forces of bacteria, bacteriophages outnumber bacteria by an order of magnitude (4, 5). Phages constitute the vast majority of marine viral communities (4 – 6) and influence nutrient cycling, host community structure, and evolution.

Marine viral community structure and diversity have been uncovered using culture-independent viral diversity surveys, such as metagenomics (7 – 12) and single-cell genomics (13 – 15). In the recent decade, enormous amounts of metagenomic data have been generated to reveal a previously unrecognized viral diversity in various marine environments (7 – 12). Meanwhile, a substantial number of novel viral genomic fragments have been assembled from marine metagenomic and viromic reads, providing valuable viral reference genomes without isolation efforts (8 – 12, 16, 17). In comparison, culture-dependent viral isolation lags far behind metagenomic studies because of the lack of laboratory marine bacterial cultures and difficulties in isolating viruses. Recently, considerable efforts have been made to isolate bacteriophages that infect ecologically important and abundant marine bacterioplankton. For example, SAR11 phages (pelagiphages), *Roseobacter* RCA phages, and SAR116 phages have been isolated and shown to have significant genomic diversity and to dominate the marine viral communities (18 – 24). Isolation of these phages has greatly facilitated the annotation and host prediction of many related metagenomic viral sequences. In addition, phage isolation not only provides infectious data but also provides experimental model systems for the investigation of phage-host interactions and phage ecological impact. Phages have pervasively mosaic genomes, resulting from complex evolutionary processes over a long period of time (25, 26). Lateral gene transfer via genetic recombination and fast mutations are the major forces that promote the genomic evolution of phages (27 – 29). Analysis of the phage genomes can help to understand phage evolution.

As an important group of methylotrophs, the OM43 clade in the family *Methylophilaceae* of *Betaproteobacteria* occurs commonly in marine environments and plays an important role in C1 metabolism and carbon cycling (30 – 37). The OM43 clade was first discovered by sequencing ribosomal RNA genes from the western Atlantic coast (30), and it was speculated to be linked to phytoplankton populations and primary productivity (33). The OM43 clade, which has coincided with phytoplankton blooms in surface water (36), can account for up to 5% of the microbial community in coastal ecosystems (30). Members of the OM43 clade are small and genomically streamlined (~1.3 Mbp) (34, 35, 37). To date, there are two known OM43 subgroups (37), the H-RS cluster and the HTCC2181 cluster. OM43 bacteria are difficult to culture in laboratories owing to their sensitivity to slight biochemical variations in seawater (34).

To date, only three OM43 phage isolates have been reported, phage Venkman (EXVC282S), isolated from the western English Channel on OM43 strain H5P1 (23), phage MEP301, isolated from the Bohai Sea on OM43 strain HTCC2181 (38), and phage Melnitz (EXVC044M), isolated from the western English Channel and Sargasso Sea on OM43 strain H5P1 (39). These three OM43 phages were classified into three distinct viral groups. Venkman and MEP301 both have an icosahedral head and a short tail, belonging to two distinct viral groups (23, 38). Melnitz has a myovirus morphotype and is most closely related to pelagiphage Mosig and HTVC008M (39). Metagenomic analysis showed that most OM43 phages were correlated with temperature, having a higher relative abundance in cold waters (38, 39).

To broaden the understanding of marine OM43 phages, a novel OM43 strain, FZCC0133, was used as a host to isolate OM43 phages. FZCC0133 belongs to a novel OM43 subgroup, separated from the H-RS cluster and HTCC2181 cluster. Two phages that infect FZCC0133 were isolated and designated as MEP401 and MEP402. A metagenomic mining was performed to identify metagenomic viral genomes (MVGs) related to MEP401 and MEP402. Genomic analyses revealed that MEP401, MEP402, and all the recovered MVGs represent a novel phage subfamily in the family *Zobellviridae* that can be separated into two genus-level groups. A metagenomic recruitment analysis was also performed to illustrate the global distribution patterns of these phages.

## RESULTS

### Host strain

The OM43 strain FZCC0133 was isolated in 2017, from the coastal water of Pingtan Island, Fujian, China (25°26'N, 119°47'E), using the dilution-to-extinction method. 16S rRNA gene analysis showed that FZCC0133 is more closely related to OM43 strain H5P1 (98.90% 16S rRNA gene sequence identity) than H-RS and HTCC2181 (96.72% and 97.64% 16S rRNA gene sequence identity, respectively). Phylogenetic analysis based on 16S rRNA gene sequences showed that FZCC0133 falls within the OM43 clade and is distinct from the known H-RS and HTCC2181 clusters. This result suggests that FZCC0133 and H5P1 belong to a new cluster within the OM43 clade (Fig. S1).

### Isolation and biological features of MEP401 and MEP402

**Fig 1:**
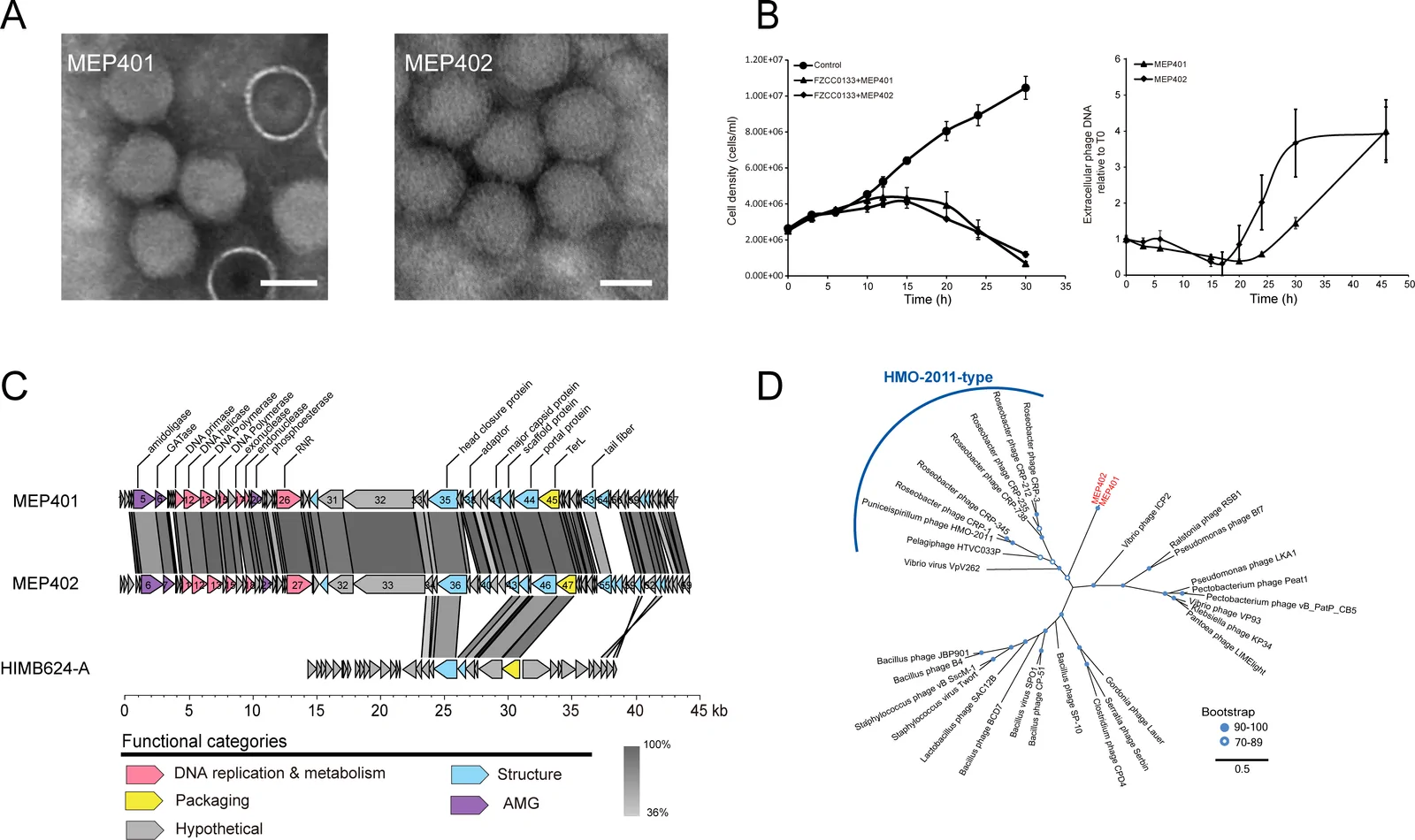
General biological and genomic characteristics of MEP401 and MEP402. (**A**) Transmission electron microscopy images of MEP401 and MEP402. (**B**) Left panel: Growth curves of host FZCC0133 infected with the two OM43 phages. Right panel: Growth curves of the two OM43 phages on exponential cultures of FZCC0133. (**C**) Genomic alignment and comparison of MEP401, MEP402, and HIMB624-A. Predicted open reading frames (ORFs) are represented by arrows, and the direction of transcription is indicated by the direction of the arrow. The numbers inside the arrows indicate ORF numbers. ORFs annotated with known functions are marked using distinct colors according to their functions. (**D**) Maximum-likelihood phylogenetic tree of DNA polymerase constructed with conserved polymerase domains. MEP401 and MEP402 are shown in red.

The OM43 phage MEP401 was isolated from the coastal surface water of Yantai, Bohai Sea, China (37°28'N, 121°29'E), whereas MEP402 was isolated from the coastal surface water of Osaka Bay, Japan (34°27'N, 135°21'E). The transmission electron microscopy (TEM) images showed that the two phages have an icosahedral capsid of 62 ± 2 nm in diameter with an obscure short tail (Fig. 1A). The morphological characteristics of these two phages suggest that they belong to the order *Caudoviricetes*.

Infections of FZCC0133 cell cultures by both MEP401 and MEP402 caused sharp declines in host numbers and produced phage particles (Fig. 1B). The latent periods of MEP401 and MEP402 are both approximately 17–20 hours.

### General genome characteristics of MEP401 and MEP402

Genome sequencing revealed that MEP401 and MEP402 have dsDNA genomes of 42,987 bp and 43,738 bp in size, respectively (Table 1). The G + C content of both phage genomes is approximately 34%, which is similar to that of their host FZCC0133 (33.1%). Their G + C content is slightly higher than that of the first OM43 phage Venkman (31.9%) (23) but lower than those of the previously reported OM43 phages MEP301 (44.4%) (38) and Melnitz (37.6%) (39). MEP401 and MEP402 encode 67 and 69 open reading frames (ORFs), respectively. According to the direction of transcription, the two genomes can be divided into two reverse direction genome units (Fig. 1C). ORFs 1–28 in MEP401 and ORFs 1–29 in MEP402 have a reverse transcription direction compared with that of the remaining ORFs in both genomes. A total of 62 ORFs are shared between these two phages with high homology (43%–100% amino acid identity) (Fig. 1C). Genome comparison revealed that MEP401 and MEP402 share high sequence similarity, with 92.19% average nucleotide identity (ANI) and 85.15% average amino acid sequence identity (AAI). Since 95% ANI threshold is used as one of the standards for phage species level demarcation (40, 41), MEP401 and MEP402 can be considered as distinct phage species. Further genomic analysis showed that they do not display substantial genome similarity with any cultured phages and therefore belong to a novel phage group. Approximately 31% of the identified ORFs in both genomes could be assigned putative biological functions based on the sequence similarities and conserved domains. Genes responsible for phage DNA replication and metabolism, structure and DNA packaging, and cell lysis were identified in MEP401 and MEP402.

**TABLE 1:** General features of two OM43 phages isolated in this study

Phage	Original host	Source water	Depth	Latitude	Longitude	Genome size (bp)	No. of ORFs	% G + C	Accession number
MEP401	FZCC0133	Yantai, Bohai Sea	Surface	N37°28'	E121°29'	42,987	67	34.4	OP830906
MEP402	FZCC0133	Osaka Bay, Japan	Surface	N34°27'	E135°21'	43,738	69	34.3	OP830907

In the DNA replication region, both genomes contain genes encoding DNA helicase, DNA primase, DNA polymerase (DNAP), endonuclease, exonuclease, ribonucleoside-triphosphate reductase (RNR), and a few proteins with unknown functions (Fig. 1C). DNAPs in MEP401 and MEP402 are encoded by two ORFs (ORF13 and ORF15 in both genomes) (Fig. 1C), which are segmented by a small unknown protein (ORF14 in both genomes). ORF13 in MEP401 and MEP402 contain the 3′−5′ exonuclease domain (PF01612) and the *N*-terminal DNA polymerase A domain (PF00476), whereas ORF15 in both phages contain the *C*-terminal DNA polymerase A domain (PF00476). In many phage genomes, group I introns are inserted into phage DNAP genes (42 – 44). Thus, it was possible that DNAP genes in MEP401 and MEP402 both phages were interrupted by an intron.

The HMO-2011-type phages and *Vibrio* phage ICP2 appear to be the closest relatives of MEP401 and MEP402 based on sequence analysis. The DNAPs of MEP401 and MEP402 share 24.8%–31.2% amino acid identities with those in HMO-2011-type phages. However, DNAPs in MEP401 and MEP402 do not possess the unusual domain structure of typical HMO-2011-type DNAPs (19, 24) and only share a few genes with HMO-2011-type phages. These results suggest that MEP401 and MEP402 are distinct from HMO-2011-type phages. The DNAPs of MEP401 and MEP402 share 28.0% and 28.2% amino acid identities with that in phage ICP2, respectively. In addition, both MEP401 and MEP402 possess 13 other genes that are homologous to those in ICP2, suggesting that they may have evolutionary relatedness with ICP2. Phylogenetic analysis of the DNA polymerase family A domain confirmed that MEP401 and MEP402 are distinct from other known phages, representing a novel phage group (Fig. 1D). The DNA primases of MEP401 and MEP402 share weak homology with those in *Ralstonia* phage RSB1 (NC_011201) (31.4% amino acid identity) and *Burkholderia* phage Bp-AMP4 (HG796221) (30.5% amino acid identity). The DNA helicases of MEP401 and MEP402 share sequence similarity with that in *Burkholderia* phage JG068 (NC_022916) with approximately 40% amino acid identity. The DNA endonucleases of MEP401 and MEP402 are similar to those in *Cyanophage* KBS-S-1A (JF974297) (~43% amino acid identity). Their DNA exonucleases share approximately 38% amino acid identity with that of *Roseobacter* phage CRP-5 (MK613347).

In their structural and packaging regions, MEP401 and MEP402 have genes predicted to be involved in phage morphogenesis and packaging, including genes encoding major capsid protein (MCP), scaffold protein, tail protein, portal protein, and terminase large subunit (TerL). Some of the structural genes in MEP401 and MEP402 share similarities with those in other phages. For example, their MCPs are homologous to that in *Vibrio* virus VPMCC5 (OL676770) with 53.4% amino acid identity. Their scaffold proteins share 38.2% amino acid identity with that in *Salinivibrio* phage CW02 (NC_019540). The TerLs of MEP401 and MEP402 share approximately 54.0% amino acid identity with that of *Lentibacter* phage vB_LenP_ICBM2 (NC_048671). Their portal proteins are similar to that in *Citrobacter* phage CVT22 (51.2% amino acid identity).

When searching against the NCBI database, we noticed that MEP401 and MEP402 share high similarity with an unfinished OM43 phage HIM624-A (AFB70783.1), which infects *Methylophilacea* HIMB624. The partial genome of HIM624-A is 23,493 bp in size, encoding 35 ORFs. A total of 14 ORFs in HIM624-A have homologs in MEP401 and MEP402, with 22%–83% amino acid identity (Fig. 1C). The partial genome of HIM624-A only covers the structural and packaging module and does not contain any replication-related genes. This analysis suggests that HIMB624-A may belong to the MEP401-type phage group.

### Identification of MVGs related to MEP401 and MEP402 from the metagenomic data sets

To expand our knowledge of the genomic diversity of phages related to MEP401 and MEP402, a search was performed to retrieve MVGs from environmental metagenomes. After the workflow for MVGs retrieval, a total of 99 MVGs (>50% genome completeness) from various oceanic stations were extracted for further analysis. Most of these MVGs originated from surface waters (0–200 m) of polar and estuarine regions (Table S1).

**Fig 2:**
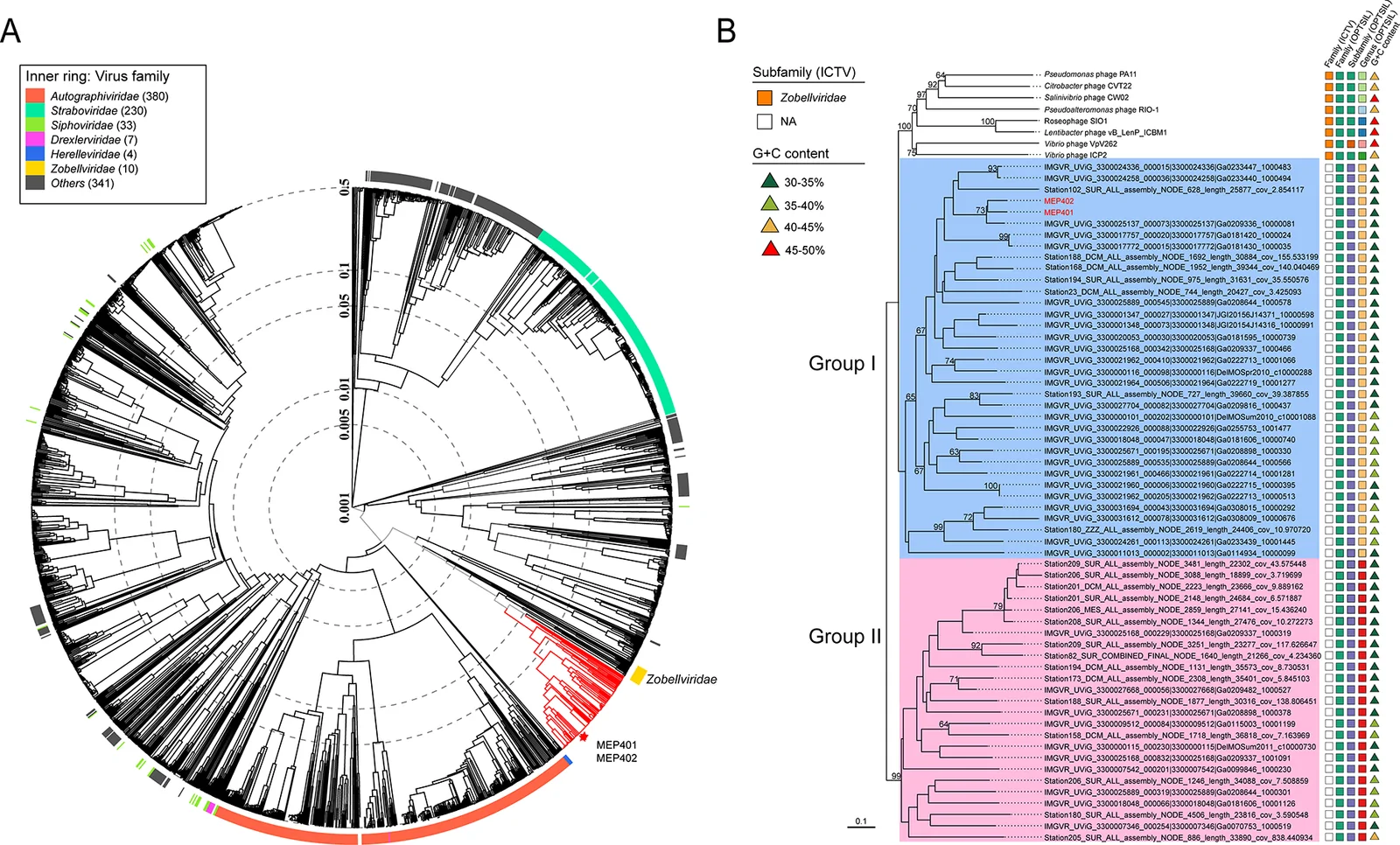
(**A**) Viral proteomic tree for MEP401, MEP402, and all retrieved MVGs. The tree was generated using ViPTree against all prokaryotic dsDNA viruses. Only the genomes related to the analyzed phages are shown. Red branches indicate MEP401, MEP402, and all retrieved MVGs. MEP401 and MEP402 are indicated with red asterisks. (**B**) Whole-genome-based phylogenetic tree of MEP401-related phages at the amino acid level. The tree was constructed by VICTOR. All phages were clusters at 95% ANI, and the longest genomes in each cluster were used for VICTOR analysis. The predicted OPTSIL taxon is shown, and the G + C content of each genome is indicated.

A phylogenetic tree based on the viral proteome was constructed by ViPTree. MEP401, MEP402, and the retrieved MVGs were located adjacent to phages of the family *Zobellviridae* in the ViPTree, suggesting their evolutionary relatedness with members of this family. Whole genome-based VICTOR phylogeny analysis showed that MEP401, MEP402, and all retrieved MVGs belong to the family *Zobellviridae* but form a distinct subfamily. The subfamily can be further divided into two groups, with 64 MVGs clustering with MEP401 and MEP402 (group I), and the remaining 35 MVGs forming a separate group (group II) (Fig. 2). All group I MVGs were classified into the same genus with MEP401 and MEP402 using VICTOR (Fig. 2). Host prediction using the RaFAH tool also assigned OM43 bacteria as potential hosts of most group I MVGs (Table S1). These group I MVGs range in size from 20.4 to 47.7 kb (50.94%–100% genome completeness), encoding 27–73 ORFs, and their G + C content ranges from 31.1% to 38.6%. The 35 MVGs in group II were assigned into a different genus by VICTOR analysis (Fig. 2). MVGs in this group have a genome size ranging from 18.1 to 44.7 kb (50.84%–100% genome completeness), with a G + C content ranging from 32.3% to 46.9%. Host prediction using the RaFAH tool suggested that most group II phages also infect OM43 bacteria (Table S1).

### Conserved genomic structure and gene content variation

**Fig 3:**
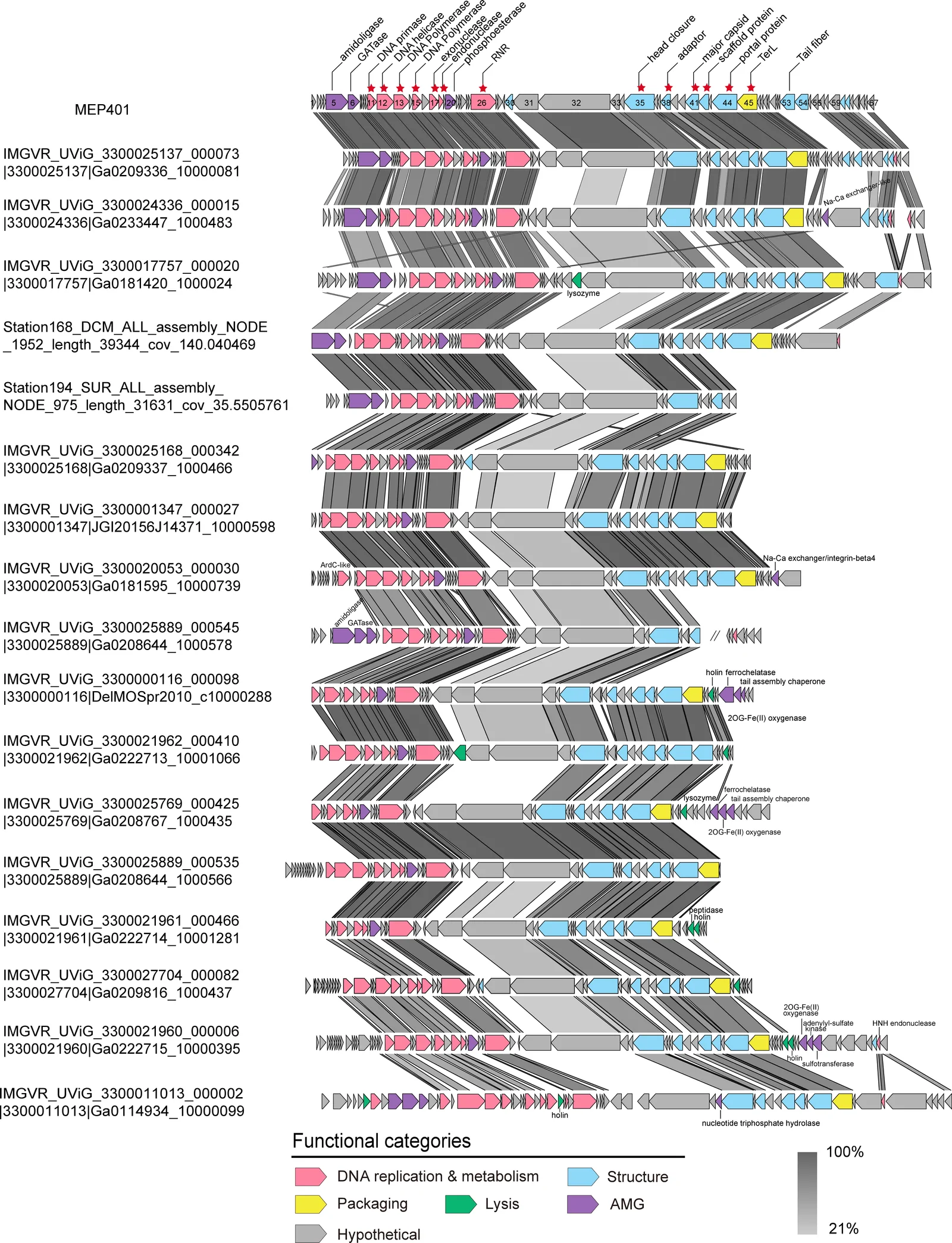
Alignment and comparison of genomes of representative group I phages showing the genomic synteny of this group. Open reading frames (ORFs) are represented by arrows, and the direction of transcription is indicated by the arrow directions. The numbers inside the arrows indicate ORF numbers. ORFs annotated with known functions are marked using distinct colors according to their functions. Core genes are indicated with red asterisks. The color of the shading connecting homologous genes indicates the level of amino acid identity between the genes. RNR, ribonucleoside-triphosphate reductase; TerL, terminase large subunit; GATase, glutamine amidotransferase.

Pangenome analysis identified 328 orthologous protein groups (containing two or more members), of which 91 protein groups could be assigned biological functions (Table S2). Genomic comparison revealed that genome synteny was well conserved across all group I genomes, with few genomic rearrangements (Fig. 3). The functional module structure of all group I MVGs is similar to that of MEP401 and MEP402, and they have significant sequence similarity in the DNA replication and metabolism module and in the structural and DNA packaging module. All group I MVGs were also referred to as MEP401-type MVGs. Split DNA polymerases were also observed in most group I MVGs (Fig. 3). Core genome analysis based on the complete group I genomes identified 13 core genes that were shared by all the complete group I genomes (Fig. 3). These core genes predominantly encode proteins related to phage DNA replication, phage development, and DNA packaging, suggesting that group I phages employ similar overall replication and propagation processes.

**Fig 4:**
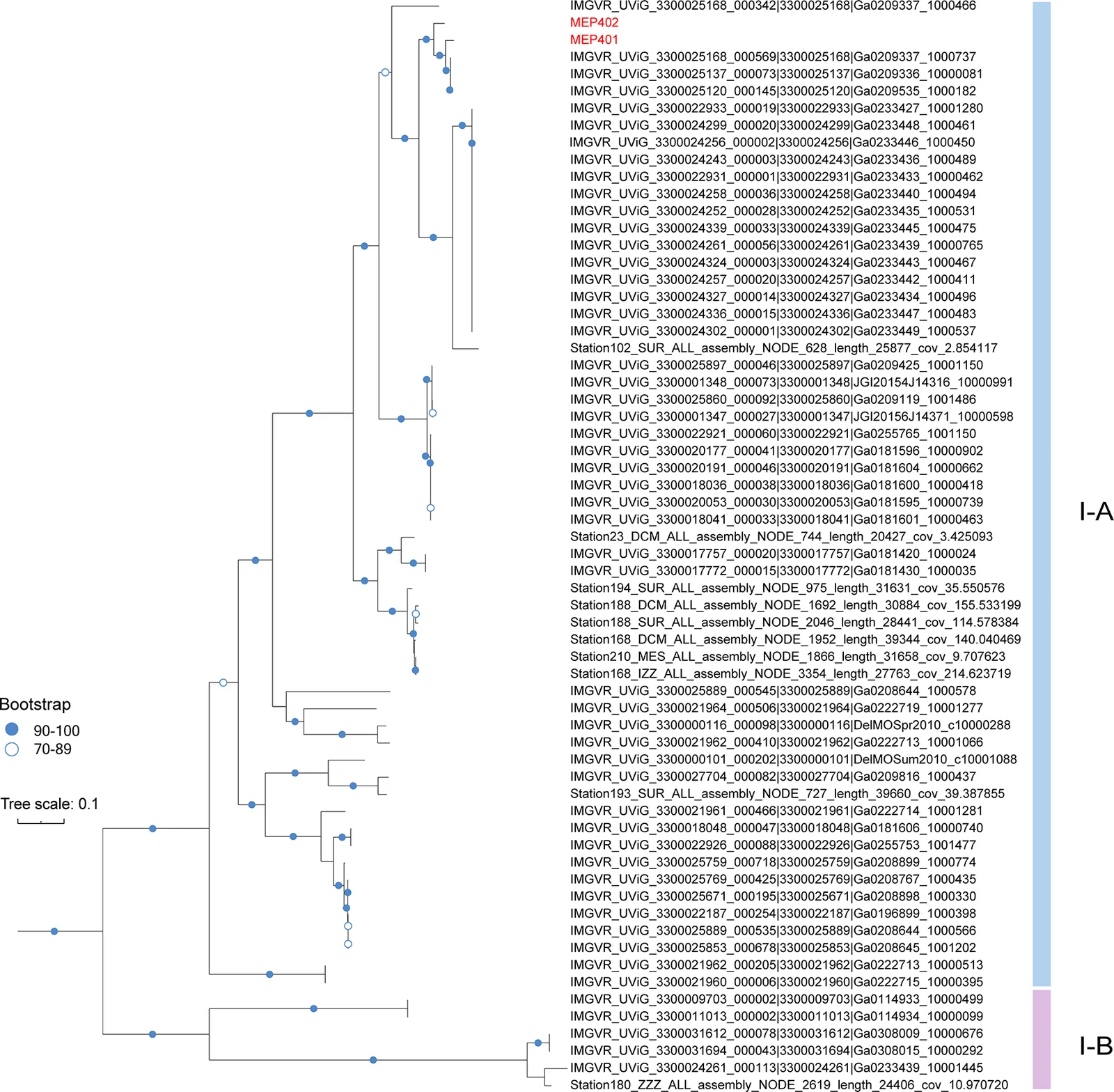
A maximum-likelihood tree was constructed using the concatenated sequences of 10 core genes in group I genomes. The scale bar indicates the number of amino acid substitutions per site.

Phylogenomic analysis based on the concatenated sequences of 10 selective core genes was performed to resolve the evolutionary relationship among group I phages. The tree topology was similar to that of the VICTOR analysis. The phylogenomic tree showed that group I phages are diverse and can be separated into two well-supported subgroups (I-A and I-B) (Fig. 4). Subgroup I-A contains MEP401, MEP402, and 56 MVGs from various oceanic regions, while subgroup I-B only contains six MVGs. Subgroups I-A and I-B belong to the same genus according to the VICTOR analysis (Fig. 2B).

**Fig 5:**
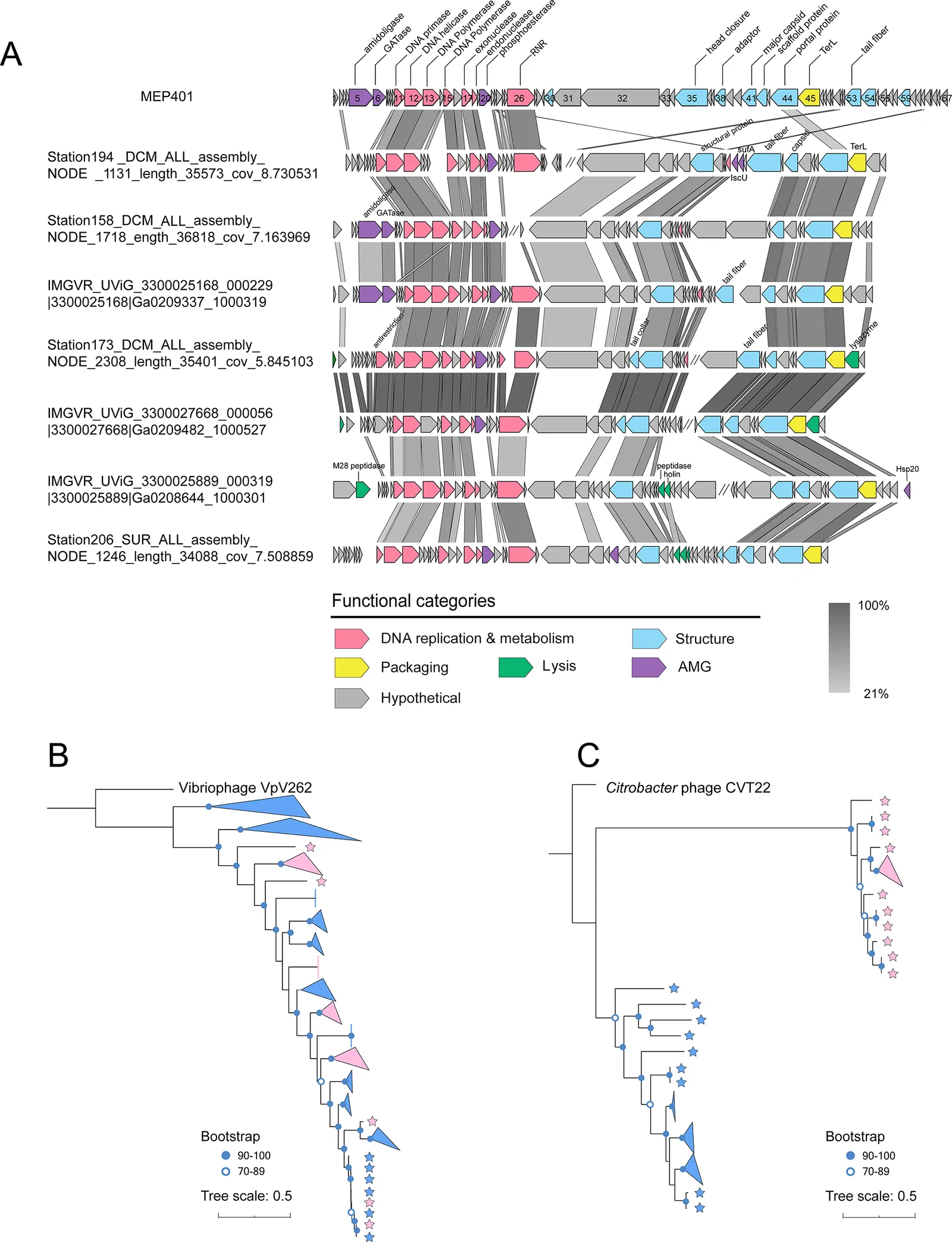
(**A**) Genomic organization and comparison of group II genomes showing the combination of the MEP401-type DNA replication module and a distinct type of morphogenesis and packaging module. Open reading frames (ORFs) are represented by arrows, and the direction of transcription is indicated by the arrow directions. The numbers inside the arrows indicate ORF numbers. ORFs annotated with known functions are marked using distinct colors according to their functions. The color of the shading connecting homologous genes indicates the level of amino acid identity between the genes. RNR, ribonucleoside-triphosphate reductase; TerL, terminase large subunit; IsuC, Fe-S cluster assembly scaffold protein; SufA, Fe-S cluster assembly scaffold protein. (**B**) Maximum-likelihood phylogenetic tree of all group I and group II phages based on DNA polymerase sequences. (**C**) Phylogenetic tree constructed based on the large terminase (TerL) sequences. Group I and group II phages are indicated by blue and pink asterisks, respectively.

Genomic comparisons revealed that group II MVGs also have a DNA replication module similar to those in group I phages (Fig. 5A). In contrast, only a few small genes in the morphogenesis and packaging modules of group II MVGs have homologs in group I genomes. Their MCPs and TerLs are very distantly related to those in group I MVGs but more closely related to those in Cyanophage PP (30%–36.5% amino acid identity) and *Pseudomonas* phage hairong (37%–47.8% amino acid identity). The conserved DNAP and TerL sequences were used for phylogenetic analyses. DNAP phylogeny demonstrated that all group I and II phages cluster together and that DNAPs from group II did not show a good separation from those in group I (Fig. 5B), suggesting that these two groups share a common ancestor. However, the TerL phylogeny showed that group II phages are distinct from group I phages (Fig. 5C), suggesting that they may have arisen from recombination between two different phage ancestors.

### Metabolic capacity

Auxiliary metabolic genes (AMGs) are phage-encoded metabolic genes that are highly similar to their host homologs. Several AMGs that are potentially involved in diverse metabolic processes have been identified in group I and II genomes. In the DNA replication module, 73 MVGs were found to possess calcineurin-like metallophosphoesterase genes (MPEs, PF00149), sharing homology with those in *Pseudomonas* phage VCM (43.2%–47.8% amino acid identity). This gene was also identified in OM43 phage. Genes encoding amidoligase (PF12224) and glutamine amidotransferase (GATase) (PF13522) were identified in MEP401, MEP402, and over 30 MVGs. Six MVGs from groups I and II contain genes encoding the 2-Oxoglutarate and Fe(II)-dependent oxygenase (2OG-Fe(II) oxygenase) superfamily (PF13640), sharing 29.2%–38.3% amino acid identity with that in *Synechococcus* phage ACG-2014f. Genes encoding the Fe-S cluster assembly scaffold protein (*sufA*, PF01521) were identified in two group I MVGs. These genes have been previously identified in HMO-2011-type MVGs (24). Two MVGs encode the sulfotransferase family protein (Sulfotransfer_3 domain, PF13469), sharing similarity with that in *Synechococcus* phage S-SRM01 (33.2% amino acid identity).

### Distribution in the global ocean

**Fig 6:**
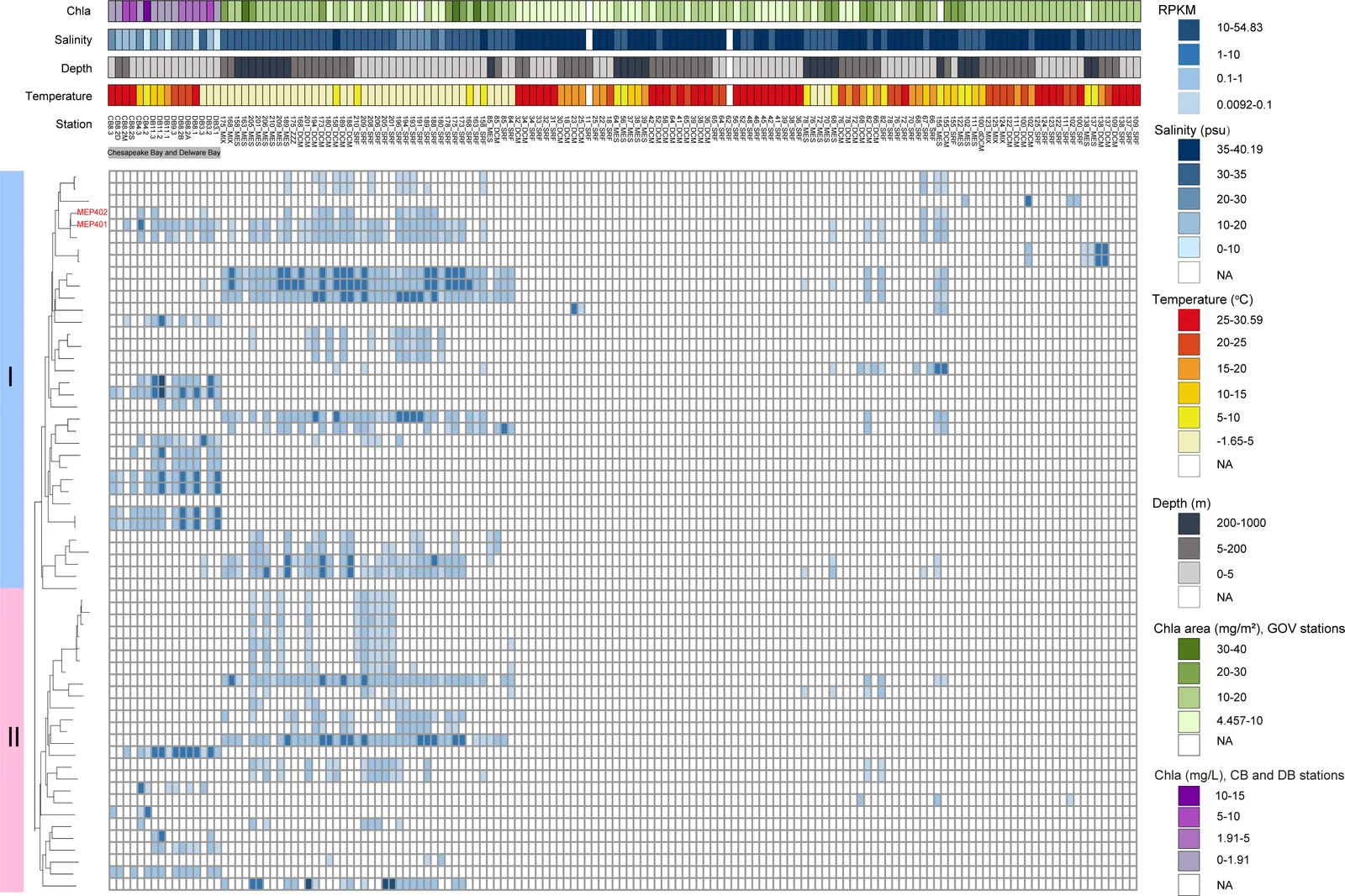
Heatmap displaying the RPKM of group I and group II phage in different marine viromic data sets. Normalized relative abundance is depicted as reads recruited per kilobase of contig per million reads (RPKM). DCM, deep chlorophyll maximum; MESO, mesopelagic; SRF, surface; MIX, bottom of mixed layer; DB, Delware Bay; CB, Chesapeake Bay; NA, not available.

To demonstrate the biogeographical patterns of phages in these two groups, their presence and RPKM (reads per kilobase pair of genomes per million reads) at various oceanic stations were analyzed by mapping the reads from 147 viromes to each phage genome (≥95% nucleotide identity). Generally, these phages were exclusively detected in the surface and mesopelagic waters (0–1,000 m), with varying RPKM values (Fig. 6). Among all analyzed phages, many were more prevalent and abundant in colder regions (Fig. 6). These oceanic regions also exhibit higher chlorophyll values and lower salinities. The linear regression analysis showed significant negative correlation between the RPKM values of some phages and temperature (*P* < 0.05) (Table S3). In addition, these phages were mainly found in oceanic regions and were rarely found in estuarine stations. We found that MEP401, MEP402, and several MVGs closely related to MEP401 and MEP402 exhibited a relatively wider distribution compared with other MVGs. They were detected not only in cold regions but also in Chesapeake Bay and Delaware Bay stations with high temperature and lower salinity (<20 psu). Furthermore, we found that most MVGs that were not correlated with temperature originated from various estuarine stations and were exclusively distributed in estuarine environments (Fig. 6).

## DISCUSSION

The marine OM43 clade is one of the most abundant bacterial groups in the ocean. Bacteria in this group have diverse metabolic profiles and play important roles in the metabolism of C1 compounds. Phages that infect marine OM43 bacteria are poorly characterized. In this study, two novel OM43 phages, MEP401 and MEP402, infecting OM43 strain FZCC0133 were isolated. Genomes of these two OM43 phages share high sequence identity with each other but are distinct from other known phages.

Genes related to DNA replication and metabolism, phage structure, and DNA packaging in the two OM43 phages were found sharing sequence identity with some known phages, most of which are *Zobellviridae* phages, implying that MEP401 and MEP402 are evolutionary related to *Zobellviridae* phages. The DNAP genes are responsible for phage genome replication and play an important role in shaping the evolutionarily history and fitness of the phages (45 – 47). DNAPs of both MEP401-like phages split into two genes with a small unknown ORF inserted. Homing endonucleases-encoding introns were commonly found in many phage genomes (42 – 44, 48, 49). The introns interrupt various phage genes and can function as self-splicing ribozymes (42 – 44, 48, 49). It is possible that DNAP genes in MEP401 and MEP402 were also interrupted by an intron. However, in our case, the two inserted ORFs do not show homology to any known gene products. Whether these small ORFs are self-splicing introns is unknown. Their functions remain to be further explored.

Metagenomic mining identified a total of 99 MVGs related to MEP401 and MEP402. Phylogenomic analyses revealed that MEP401, MEP402, and all retrieved MVGs represent a novel phage subfamily in the family *Zobellviridae*. They are distinct from other known members of this family and can further be separated into two genus-level groups (group I and group II). Genome comparison analysis revealed that group I and group II members have similar DNA replication modules, but their morphogenesis and packaging modules are distinct. These findings suggest that phages in group I and group II have a close evolutionary relationship and have undergone genome recombination events. Genetic recombination between phage populations can produce novel combinations of genomic modules and is the main driving force of phage evolution (26 – 29, 50 – 53). The genomic characteristics and phylogenetic analyses of group I and group II phages suggest that genome recombination is instrumental in the genome evolution and diversification of OM43 phages.

AMGs are a class of phage-encoded metabolic that have potential roles in regulating host metabolism (3). A set of AMGs were identified from group I and II genomes, including genes encoding MPEs, amidoligase, GATase, 2OG-Fe(II) oxygenase, SufA, and Sulfotransferse. MPEs exhibit hydrolase activity against a wide range of phosphorylated substrates and are common in bacterial and archaeal genomes (54 – 56). Phage-encoded MPEs have been identified in many phage genomes (56, 57). A previous study has suggested that *Mycobacterium* virus D29-encoded MPEs can negatively regulate the growth of bacteriophages and bacteria (56). The function of phage-encoded MPEs in OM43 phages requires further investigation. Amidoligase and GATase have been suggested to be involved in modifying the cell wall and thus preventing superinfection by other phages in *Escherichia* phage phiEco32 (58) and suggested to be involved in the synthesis of secondary metabolites and the interactions between phage and hosts in mycobacteriophage Marvin (59). The prevalence of genes encoding these two proteins suggests that they may play a vital role in these phages; however, their specific functions remain to be explored. The 2OG-Fe(II) oxygenase superfamily is typically involved in the oxidation of organic substrate using a dioxygen molecule, which is mainly responsible for protein modification, nucleic acid repair and/or modification, and fatty acid metabolism (60, 61). Fe-S cluster participates in a wide variety of cellular biological processes (62). SufA is a scaffold protein for Fe-S cluster assembly. The discovery of SufA genes in two group I MVGs suggests that these phages may play important roles in Fe-S cluster biogenesis and function. Sulfotransferases are responsible for transferring a sulfate group from 3′-phosphoadenylyl sulfate to a wide range of substrates and therefore have many functions (63). However, the biological function of sulfotransferases in phage genomes remains unclear.

Read-mapping analysis indicated that many phages in these two groups were more prevalent and abundant in cold regions and showed patterns that were similar to some previously reported OM43 phages (23, 38), implying that their hosts were more prevalent in cold waters. Currently, the OM43 lineage has two identified ecotypic clusters, with the H-RS cluster more abundant in low-chlorophyll a and/or warm water, while the HTCC2181 cluster is more abundant in lower temperature but higher-productivity waters (37). Furthermore, in a previous study, OM43 phages displayed two distinct distribution patterns, similar to the distributions of two known OM43 clusters (37). In this study, we found several MVGs have a distribution pattern that is distinct from the distributions of previously reported OM43 phages. These MVGs were exclusively prevalent in estuarine stations. Considering that the distribution of phages is broadly correlated with the distribution of their hosts, it is possible that these MVGs infect an OM43 cluster that is specifically adapted to estuarine environments.

### Conclusion

In this study, two new phages (MEP401 and MEP402) that infect the marine OM43 strain FZCC0133 were isolated and sequenced. In addition, 99 MVGs related to MEP401 and MEP402 were identified using a metagenomics-based mining analysis. Through comparative genomic and phylogenomic analyses, these phages were shown to represent a novel phage subfamily that can be separated into two phage groups with distinct types of morphogenesis and packaging modules. These results further support the idea that genetic recombination plays an important role in the generation of phage genetic diversity. Furthermore, metagenomic mapping analysis revealed the ubiquity and distinct distribution patterns among the members of these two phage groups. Overall, our study provides novel insights into the diversity and biogeography of phages infecting marine OM43 bacteria and establishes models as valuable tools for evaluating the ecological roles of viruses in marine environments.

## MATERIALS AND METHODS

### Cultivation, purification, and phylogenetic analysis of OM43 strain FZCC0133

The OM43 strain FZCC0133 was isolated in May 2017 from the coastal waters of Pingtan Island in China (N25°26′, E119°47′) using the dilution-to-extinction method with low-nutrient medium (64). FZCC0133 was grown in a sterilized natural seawater–based medium with 100 µM methanol, 1 mM NH_4_Cl, 100 µM KH_2_PO_4_, 1 µM FeCl_3_, and a vitamin mixture (65). The FZCC0133 cultures were incubated at 23°C in the dark without shaking. The 16S rRNA gene of FZCC0133 was amplified using PCR with the universal primers 16S-27F and 16S-1492R (66). The 16S rRNA gene sequence of the strain was obtained by Sanger sequencing and assembled using Chro-masPro (Technelysium Pty. Ltd., Tewantin, QLD, Australia). A phylogenetic tree based on 16S rRNA gene sequences was constructed using IQ-TREE v1.6.12 (67) with 1,000 bootstrap replicates.

### Source waters and OM43 phage isolation

The water samples used to isolate OM43 phages were collected from two different coastal stations: Yantai coast, Bohai Sea, China (37°28'N, 121°29'E), and Osaka Bay, Japan (34°27′N, 135°21′E). The seawater samples were filtered through 0.1-µm-pore-size filters (Pall Life Sciences) to remove nonviral components and stored at 4°C prior to use. Details of phage isolation have been previously described (18, 20, 22). Briefly, filtered seawater samples were inoculated with exponential phase FZCC0133 culture. A Guava EasyCyte flow cytometer (Merck Millipore, Billerica, MA) was used to monitor cell growth. For cultures that displayed a significant cell decrease, the presence of phage particles was confirmed using epifluorescence microscopy (68) Purified phage clones were obtained using the dilution-to-extinction method (18, 20), and phage purity was verified using genome sequencing.

### Transmission electron microscopy and growth experiments

The morphology of the isolated OM43 phages was observed using TEM. The OM43 phages lysate was filtered through a 0.1-µm filter, concentrated using Amicon Ultra centrifugal filters (30 kDa; Merck Millipore), and centrifuged by ultracentrifugation (Beckman Coulter, USA) at 50,000 × *g* for 2 hours. A drop of the concentrated phage sample was placed on a copper TEM grid and subsequently dried in the air. The grid was stained for 2 minutes with 2% uranyl acetate and then observed under a Hitachi TEM at a voltage of 80 kV. The growth experiments were performed as described previously (18). For the growth curves of hosts, exponentially growing cultures of FZCC0133 were inoculated with the phages at a phage-to-host ratio of ~3. Cultures without the addition of phages were set as controls. Cell counts were determined using a flow cytometer. For the growth curves of phages, exponentially growing cultures of FZCC0133 were inoculated with the phages at a phage-to-host ratio of ~0.1. After the addition of the phages, an aliquot of the cell suspension was collected for phage enumeration from each culture every 2–6 hours for 30 hours, and the relative abundance of phage particles was quantified by quantitative PCR (qPCR). Two pairs of specific qPCR primers (Table S4) were designed using Primer-BLAST tool, targeting the DNAP sequences of the two OM43 phages. Phage lysates from different time points were used as templates for qPCR. Reaction was performed using the Taq Pro Universal SYBR qPCR Master Mix kit (Vazyme, Nanjing, China) with three biological replicates and three technical repeats. qPCR conditions were as follows: initial denaturation at 95°C for 3 minutes, followed by 40 cycles of 95°C for 5 seconds, 60°C for 15 seconds.

### Phage concentration, DNA extraction, and genome sequencing

Each phage lysate (150 mL) was filtered through 0.1-µm filters to remove cell debris and then concentrated to approximately 300 µL using Amicon Ultra Centrifugal Filters (30 kDa; Merck Millipore) and Nanosep Centrifugal Devices (30 kDa; Pall Life Sciences). Phage genomic DNA was extracted using a Blood & Tissue Kit (Qiagen, Hilden, Germany). Whole-genome sequencing of MEP401 and MEP402 were conducted using the Illumina HiSeq 2500 platform (paired-end technology 2 × 150 bp). Quality-filtering, trimming, and *de novo* assembly were performed using the CLC Genomic Workbench v11.0.1 (Qiagen, Hilden, Germany) with default settings. Gap closing of the phage genomes was performed using Sanger sequencing of PCR products covering the gap areas.

### Metagenomic retrieval of MVGs related to MEP401 and MEP402

For our analyses, marine MVGs reconstructed from the IMG/VR v3 database (17), Global Ocean Viromes (GOV and GOV 2.0) (11, 12), MedDCM fosmid library (8), Station ALOHA assembly free virus genomes (10), ALOHA 2.0 viromic database (9), 78 marine viromes from Metavir (69, 70), and 34 viral fosmids obtained from the oxic surface and oxygen-starved basin waters of Saanich Inlet (71) were downloaded. DNAP genes in MEP401 and MEP402 were used as baits to retrieve the related MVGs. Profile hidden Markov models (HMMs) were constructed using DNAP protein sequences using hmmbuild with default parameters (72). The HMM profiles were used to query the downloaded MVGs (≥10 kb) using the hmmSearch program (e-value ≤ 10^−3^ and score ≥50). Only matches with ≥25% aminio acid identity were considered. Of these MVGs, those containing HMO-2011-type DNAP (24) were removed. This analysis procedure retrieved 5,256 MVGs containing MEP401 DNAP homologs. The whole-genome phylogenetic analysis of these 5,265 MVGs, MEP401, MEP402, and bacterial virus genomes downloaded from NCBI-RefSeq (v96) was then performed using the GL-UVAB workflow (73) with the Dice coefficient under default settings. Taxonomic classification of phages at the subfamily level was performed according to the recommended minimum node depth of 0.0056 and the number of representatives ≥3. A total of 2,915 MVGs classified into the same phage family with MEP401 and MEP402 were used for further analysis. Protein clustering networks for taxonomic assignment were performed using vConTACT 2.0 (74) with default settings. Viral clusters were identified using ClusterONE (75) with the default parameters defined in vConTACT 2.0. The network was visualized using an edge-weighted spring embedded model in Cytoscape v3.8.0. Here, only MVGs that have genome-genome similarity score of ≥1 were considered. CheckV v0.8.1 was used for the completeness and quality estimation of these MVGs (76). MVGs with a genome completeness ≥50% were used for comparative genomic and ecological analyses.

### Genome annotation and comparative genomic analysis

The GeneMark online server (77) and Prodigal (78) were used to predict ORFs from all phage genomes. Translated ORFs were analyzed and annotated using BLASTP against the NCBI nonredundant and NCBI Refseq databases (e-value ≤10^−3^; ≥25% amino acid identity; ≥50% alignment length). The ORFs were searched against the Pfam (79) database using the HMMER web server (80) for recognizable conserved PFAM domains. For structure and function prediction, we also used the Conserved Domain Search Service of the NCBI (81) and HHpred servers (82). The phage genomes were compared and visualized using Easyfig v2.2.2 (83). OrthoFinder v2.5.2 (84) was used to identify groups of orthologous genes based on sequence similarity (BLASTP option: e-value ≤10^−3^; ≥25% identity; ≥50% alignment length). tRNA scan-SE was used to identify the tRNA genes (85). The ANI between genomes was calculated using fastANI v1.3 (86), and the AAI values were obtained using CompareM v.0.0.1 software (https://github.com/dparks1134/CompareM).

### Phylogenomic analyses

A viral proteomic tree was constructed created using ViPTree (87). Whole-genome phylogeny based on amino acid sequences was built using VICTOR (88) with the Genome-BLAST Distance Phylogeny (GBDP) method under recommended settings for prokaryotic viruses. The analyzed genomes were compared using CD-hit (89) with a nucleotide identity of ≥95% and ≥80% of the short genome (-c 0.95 -aS 0.8), and only the longest MVGs within a species cluster were retained for VICTOR analysis. Genome-based classification at the genus and family level was performed using the OPTSIL program (90). We conducted phylogenomic analyses to evaluate the evolutionary relationships between group I phages. Ten core genes were selected for phylogenomic analysis (DNA helicase, DNAP, capsid, portal, DNA primase, exonuclease, endonuclease, adaptor protein, scaffold, and TerL). Core gene sequences were aligned using MAFFT-7.455 (91) and edited using trimALv1.4.1 (92). The alignments were concatenated and a phylogenetic tree was constructed using IQ-TREE v1.6.12 (67) with 1,000 bootstrap replicates.

Phylogenetic trees of the DNAP and TerL sequences were also constructed to reveal the evolutionary relationships between the two phage groups. Amino acid sequence alignment and editing were performed using MAFFT-7.455 (91) and trimAl v1.4.1 (92), respectively. ProtTest3.4.2 was used to evaluate the optimal model and run with IQ-TREE v1.6.12. All phylogenetic trees were visualized using Interactive Tree Of Life ITOL v.5 (93).

### Recruitment of metagenomic reads and statistical analysis

The relative abundance of MEP401, MEP402, and related MVGs were estimated using a viromic read-mapping analysis. A total of 147 data sets were used for viromic read-mapping analysis. The analyzed genomes were compared using CD-hit (89) with a nucleotide identity of ≥95% and ≥80% of the short genome (-c 0.95 -aS 0.8), and only the longest MVGs within a species cluster were retained for recruitment analysis. Viromic reads were mapped against the nonredundant set of analyzed phage genomes using coverm with a nucleotide identity of ≥95%, alignment length of >50 bp (-p bwa-mem--min-read-percent-identity 95--min-read-aligned-length 50). The relative abundances of these phages were normalized by mapped reads per kilobase pair of genomes per million reads (RPKM). Phage genomes for which <40% of the genomes were covered by recruited viromic reads in a given data set were regarded as absent and were assigned an RPKM value of 0 (23). A heatmap of the RPKM of phages was generated using the pheatmap package in R. Linear regression analysis generated using R was used to test the relationship between environmental parameters and the relative abundance of these phages.

### Host prediction

Potential hosts of MEP401-related MVGs were predicted using the RaFAH tool with default settings (94). The training and validating random forest model for RaFAH was built using 4,269 host-known phages, MEP401 and MEP402.

## Data Availability

The 16S rRNA sequence of FZCC0133 has been deposited in the GenBank database under the accession number OQ306544. The genome sequences of MEP401 and MEP402 have been deposited in the GenBank database under the accession numbers OP830906 and OP830907.

## SUPPLEMENTAL MATERIAL

Supplemental material spectrum.04942-22-s0001.pdf Fig. S1 and Tables S1, S3, and S4.

Table S2 spectrum.04942-22-s0002.xlsx Protein orthologous groups.

ASM does not own the copyrights to Supplemental Material that may be linked to, or accessed through, an article. The authors have granted ASM a non-exclusive, world-wide license to publish the Supplemental Material files. Please contact the corresponding author directly for reuse.
