# Supplementary material for: Genomic diversity and biogeographic distributions of a novel lineage of bacteriophages that infect marine OM43 bacteria
Source: Microbiol Spectr. 2023 Aug 21;11(5):e04942-22. doi: 10.1128/spectrum.04942-22 (PMC10580990; doi:10.1128/spectrum.04942-22)
Supplement: Supplemental material — Fig. S1 and Tables S1, S3, and S4. [file spectrum.04942-22-s0001.pdf]

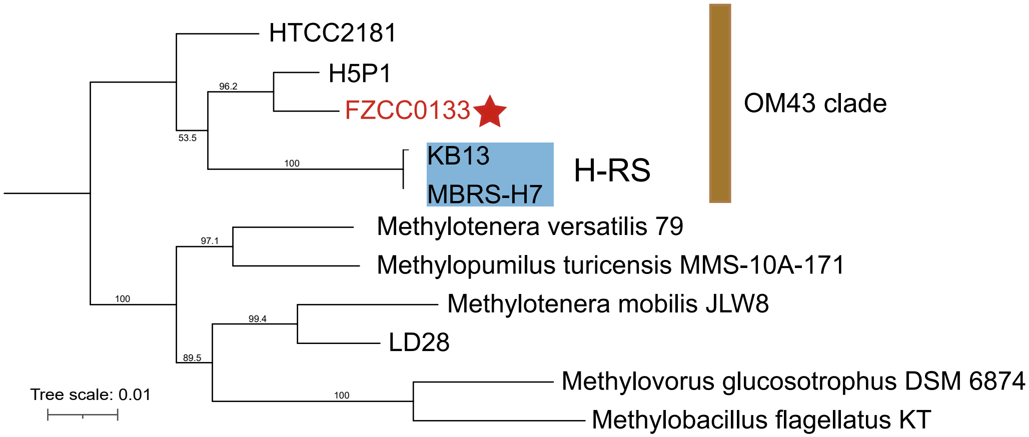

Supplemental Fig S1 Phylogenetic analysis of OM43 strains and other Methylophilaceae bacteria based on 16S rRNA gene sequences. FZCC0133 is indicated with red asterisk.

Supplementary Table 1. Summary of MEP401-type MVGs.

subgroup	MEP401-type MVGs	GC content	Length (bp)	# ORFs	Source	Depth In Meters	Genome completeness (%)	completeness_method	Predicted_Host	Predicted_Host_Score
I	IMGVR_UViG_3300025168_000342|3300025168|Ga0209337_1000466	32.19%	32687	46	Pacific Ocean	10	74.09	AAI-based (high-confidence)	OM43_clade	0.478
I	MEP401	34.37%	42987	64	Yantai, Bohai Sea, China	0	97.4	AAI-based (high-confidence)	OM43_clade	0.825
I	MEP402	34.30%	43738	67	Oaska Bay, Japan	0	99.08	AAI-based (high-confidence)	OM43_clade	0.813
I	IMGVR_UViG_3300025168_000569|3300025168|Ga0209337_1000737	33.66%	26318	33	Subarctic Pacific Ocean	10	59.65	AAI-based (high-confidence)	OM43_clade	0.353
I	IMGVR_UViG_3300025137_000073|3300025137|Ga0209336_10000081	34.18%	43213	67	Subarctic Pacific Ocean	10	97.91	AAI-based (high-confidence)	OM43_clade	0.743
I	IMGVR_UViG_3300025120_000145|3300025120|Ga0209535_1000182	34.13%	42636	66	Subarctic Pacific Ocean	10	96.6	AAI-based (high-confidence)	OM43_clade	0.737
I	IMGVR_UViG_3300022933_000019|3300022933|Ga0233427_10001280	34.05%	27809	36	Saanich Inlet, British Columbia, Canada	100	62.96	AAI-based (high-confidence)	OM43_clade	0.311
I	IMGVR_UViG_3300024299_000020|3300024299|Ga0233448_1000461	34.27%	44316	61	Saanich Inlet, British Columbia, Canada	150	100	AAI-based (high-confidence)	OM43_clade	0.545
I	IMGVR_UViG_3300024256_000002|3300024256|Ga0233446_1000450	34.26%	44168	62	Saanich Inlet, British Columbia, Canada	120	100	AAI-based (high-confidence)	OM43_clade	0.545
I	IMGVR_UViG_3300024243_000003|3300024243|Ga0233436_1000489	34.27%	44168	62	Saanich Inlet, British Columbia, Canada	150	100	AAI-based (high-confidence)	OM43_clade	0.545
I	IMGVR_UViG_3300022931_000001|3300022931|Ga0233433_10000462	34.27%	44168	62	Saanich Inlet, British Columbia, Canada	100	100	AAI-based (high-confidence)	OM43_clade	0.545
I	IMGVR_UViG_3300024258_000036|3300024258|Ga0233440_1000494	34.39%	45615	62	Saanich Inlet, British Columbia, Canada	120	100	AAI-based (high-confidence)	OM43_clade	0.545
I	IMGVR_UViG_3300024252_000028|3300024252|Ga0233435_1000531	34.30%	43999	60	Saanich Inlet, British Columbia, Canada	135	99.62	AAI-based (high-confidence)	OM43_clade	0.545
I	IMGVR_UViG_3300024339_000033|3300024339|Ga0233445_1000475	34.38%	45545	62	Saanich Inlet, British Columbia, Canada	100	100	AAI-based (high-confidence)	OM43_clade	0.545
I	IMGVR_UViG_3300024261_000056|3300024261|Ga0233439_10000765	34.27%	44692	62	Saanich Inlet, British Columbia, Canada	100	100	AAI-based (high-confidence)	OM43_clade	0.545
I	IMGVR_UViG_3300024324_000003|3300024324|Ga0233443_1000467	34.27%	44168	62	Saanich Inlet, British Columbia, Canada	200	100	AAI-based (high-confidence)	OM43_clade	0.545
I	IMGVR_UViG_3300024257_000020|3300024257|Ga0233442_1000411	34.28%	44306	61	Saanich Inlet, British Columbia, Canada	150	100	AAI-based (high-confidence)	OM43_clade	0.545

I	IMGVR_UViG_3300024327_000014|3300024327|Ga0233434_1000496	34.29%	43999	60	Saanich Inlet, British Columbia, Canada	120	99.62	AAI-based (high-confidence)	OM43_clade	0.545
I	IMGVR_UViG_3300024336_000015|3300024336|Ga0233447_1000483	34.31%	44689	64	Saanich Inlet, British Columbia, Canada	135	100	AAI-based (high-confidence)	OM43_clade	0.545
I	IMGVR_UViG_3300024302_000001|3300024302|Ga0233449_1000537	34.27%	44168	62	Saanich Inlet, British Columbia, Canada	200	100	AAI-based (high-confidence)	OM43_clade	0.545
I	Station102_SUR_ALL_assembly_NODE_628_length_25877_cov_2.854117	34.61%	25877	46	South Pacific Ocean	5	61.26	AAI-based (high-confidence)	OM43_clade	0.285
I	IMGVR_UViG_3300025897_000046|3300025897|Ga0209425_10001150	34.94%	30905	40	North Sea	1	68.97	AAI-based (high-confidence)	OM43_clade	0.502
I	IMGVR_UViG_3300001348_000073|3300001348|JGI20154J14316_100009	34.46%	25553	36	North Sea	1	62.79	AAI-based (high-confidence)	OM43_clade	0.271
I	IMGVR_UViG_3300025860_000092|3300025860|Ga0209119_1001486	34.33%	22915	30	North Sea	1	56.48	AAI-based (high-confidence)	OM43_clade	0.266
I	IMGVR_UViG_3300001347_000027|3300001347|JGI20156J14371_100005	34.78%	31273	43	North Sea	1	69.25	AAI-based (high-confidence)	OM43_clade	0.5
I	IMGVR_UViG_3300022921_000060|3300022921|Ga0255765_1001150	34.69%	33485	46	Groves Creek Marsh, Skidaway Island,	0	74.78	AAI-based (high-confidence)	OM43_clade	0.501
I	IMGVR_UViG_3300020177_000041|3300020177|Ga0181596_10000902	34.76%	30692	38	Groves Creek Marsh, Skidaway Island,	0	68.51	AAI-based (high-confidence)	OM43_clade	0.501
I	IMGVR_UViG_3300020191_000046|3300020191|Ga0181604_10000662	34.83%	31596	40	Groves Creek Marsh, Skidaway Island,	0	70.53	AAI-based (high-confidence)	OM43_clade	0.501
I	IMGVR_UViG_3300018036_000038|3300018036|Ga0181600_10000418	34.69%	32623	44	Groves Creek Marsh, Skidaway Island,	0	72.85	AAI-based (high-confidence)	OM43_clade	0.501
I	IMGVR_UViG_3300020053_000030|3300020053|Ga0181595_10000739	34.72%	36476	53	Groves Creek Marsh, Skidaway Island,	0	82.65	AAI-based (high-confidence)	OM43_clade	0.501
I	IMGVR_UViG_3300018041_000033|3300018041|Ga0181601_10000463	34.70%	32663	44	Groves Creek Marsh, Skidaway Island, Georgia	0	72.94	AAI-based (high-confidence)	OM43_clade	0.501
I	Station23_DCM_ALL_assembly_NODE_744_length_20427_cov_3.425093	31.11%	20427	29	Mediterranean Sea	55	50.94	AAI-based (high-confidence)	OM43_clade	0.177
I	IMGVR_UViG_3300017757_000020|3300017757|Ga0181420_1000024	31.96%	46479	60	oligotrophic San Pedro Time Series (SPOT) site	5	100	AAI-based (high-confidence)	OM43_clade	0.462
I	IMGVR_UViG_3300017772_000015|3300017772|Ga0181430_1000035	31.94%	46404	61	oligotrophic San Pedro Time Series (SPOT) site, San Pedro Channel, CA, USA	5	100	AAI-based (high-confidence)	OM43_clade	0.462

I	Station194_SUR_ALL_assembly_NODE_975_length_31631_cov_35.55057	33.55%	31631	42	Arctic Ocean	5	77.76	AAI-based (high-confidence)	OM43_clade	0.258
I	Station188_DCM_ALL_assembly_NODE_1692_length_30884_cov_155.533	32.79%	30884	44	Arctic Ocean	17	73.95	AAI-based (high-confidence)	OM43_clade	0.261
I	Station188_SUR_ALL_assembly_NODE_2046_length_28441_cov_114.578	32.86%	28441	38	Arctic Ocean	5	66.52	AAI-based (high-confidence)	OM43_clade	0.243
I	Station168_DCM_ALL_assembly_NODE_1952_length_39344_cov_140.040	33.83%	39344	51	Arctic Ocean	40	89.13	AAI-based (high-confidence)	OM43_clade	0.459
I	Station210_MES_ALL_assembly_NODE_1866_length_31658_cov_9.70762	33.51%	31658	42	Arctic Ocean	391	71.74	AAI-based (high-confidence)	OM43_clade	0.404
I	Station168_IZZ_ALL_assembly_NODE_3354_length_27763_cov_214.62371	33.23%	27763	37	Arctic Ocean	NA	62.92	AAI-based (high-confidence)	OM43_clade	0.271
I	IMGVR_UViG_3300025889_000545|3300025889|Ga0208644_1000578	32.64%	32123	43	Delaware River/Bay and Chesapeake Bay under freshwater to marine salinity gradient to study organic matter cycling in a time-series	NA	75.1	AAI-based (high-confidence)	OM43_clade	0.288
I	IMGVR_UViG_3300021964_000506|3300021964|Ga0222719_10001277	33.69%	24672	32	San Francisco Bay-Delta (SFBD), California, United States	4	59.35	AAI-based (medium-confidence)	OM43_clade	0.241
I	IMGVR_UViG_3300000116_000098|3300000116|DelMOSpr2010_c100002	33.15%	32855	46	Delaware Coast	0	72.75	AAI-based (high-confidence)	OM43_clade	0.336
I	IMGVR_UViG_3300021962_000410|3300021962|Ga0222713_10001066	33.73%	31367	43	San Francisco Bay-Delta (SFBD), California, United States	12	69.42	AAI-based (high-confidence)	OM43_clade	0.372
I	IMGVR_UViG_3300000101_000202|3300000101|DelMOSum2010_c10001088	37%	22299	34	Delaware Coast	0	52.59	AAI-based (medium-confidence)	NA	NA
I	IMGVR_UViG_3300027704_000082|3300027704|Ga0209816_1000437	33.75%	33255	52	West Antarctic Peninsula	1	73.55	AAI-based (high-confidence)	OM43_clade	0.31
I	Station193_SUR_ALL_assembly_NODE_727_length_39660_cov_39.38785	33.58%	39660	60	Arctic Ocean	5	87.62	AAI-based (high-confidence)	OM43_clade	0.321
I	IMGVR_UViG_3300021961_000466|3300021961|Ga0222714_10001281	35.22%	28368	42	San Francisco Bay-Delta (SFBD), California, United States	13	62.89	AAI-based (high-confidence)	OM43_clade	0.253
I	IMGVR_UViG_3300018048_000047|3300018048|Ga0181606_10000740	35.49%	27572	38	Groves Creek Marsh, Skidaway Island,	0	61.1	AAI-based (high-confidence)	OM43_clade	0.252

I	IMGVR_UViG_3300022926_000088|3300022926|Ga0255753_1001477	35.20%	28092	49	Groves Creek Marsh, Skidaway Island,	0	67.35	AAI-based (high-confidence)	OM43_clade	0.254
I	IMGVR_UViG_3300025759_000718|3300025759|Ga0208899_1000774	35.04%	23539	31	USA: Delaware Bay	NA	52.17	AAI-based (high-confidence)	OM43_clade	0.223
I	IMGVR_UViG_3300025769_000425|3300025769|Ga0208767_1000435	35.19%	34134	47	USA: Delaware Bay	NA	75.62	AAI-based (high-confidence)	OM43_clade	0.24
I	IMGVR_UViG_3300025671_000195|3300025671|Ga0208898_1000330	35.31%	36932	52	USA: Delaware Bay	NA	81.79	AAI-based (high-confidence)	OM43_clade	0.309
I	IMGVR_UViG_3300022187_000254|3300022187|Ga0196899_1000398	34.79%	23094	30	USA: Delaware Bay	NA	52.73	AAI-based (high-confidence)	OM43_clade	0.219
I	IMGVR_UViG_3300025889_000535|3300025889|Ga0208644_1000566	35.13%	32358	49	USA: Delaware Bay	NA	71.67	AAI-based (high-confidence)	OM43_clade	0.338
I	IMGVR_UViG_3300025853_000678|3300025853|Ga0208645_1001202	34.82%	23469	30	USA: Delaware Bay	NA	55.33	AAI-based (high-confidence)	OM43_clade	0.22
I	IMGVR_UViG_3300021962_000205|3300021962|Ga0222713_10000513	32.83%	43587	72	San Francisco Bay-Delta (SFBD), California, United States	12	100	AAI-based (high-confidence)	OM43_clade	0.435
I	IMGVR_UViG_3300021960_000006|3300021960|Ga0222715_10000395	32.82%	43390	73	San Francisco Bay-Delta (SFBD), California, United States	29	100	AAI-based (high-confidence)	OM43_clade	0.424
I	IMGVR_UViG_3300009703_000002|3300009703|Ga0114933_10000499	34.78%	47769	65	Deep subsurface microbial communities from Kolumbo volcano	495	100	AAI-based (high-confidence)	OM43_clade	0.219
I	IMGVR_UViG_3300011013_000002|3300011013|Ga0114934_10000099	34.79%	47769	63	Deep subsurface microbial communities from Kolumbo volcano	482	100	AAI-based (high-confidence)	OM43_clade	0.219
I	IMGVR_UViG_3300031612_000078|3300031612|Ga0308009_10000676	36.68%	23180	35	Antarctic Ocean	5	52.83	AAI-based (medium-confidence)	OM43_clade	0.141
I	IMGVR_UViG_3300031694_000043|3300031694|Ga0308015_10000292	36.62%	24403	28	Antarctic Ocean	37	53.59	AAI-based (medium-confidence)	OM43_clade	0.151
I	IMGVR_UViG_3300024261_000113|3300024261|Ga0233439_10001445	38.64%	30295	39	Saanich Inlet, British Columbia, Canada	100	66.18	AAI-based (medium-confidence)	OM43_clade	0.191

I	Station180_ZZZ_ALL_assembly_NODE_2619_length_24406_cov_10.970720	37.22%	24406	27	Arctic Ocean	NA	54.64	AAI-based (medium-confidence)	OM43_clade	0.155
II	Station209_SUR_ALL_assembly_NODE_3481_length_22302_cov_43.5754	33.04%	22302	35	Arctic Ocean	5	59.77	AAI-based (high-confidence)	OM43_clade	0.225
II	Station206_SUR_ALL_assembly_NODE_3088_length_18899_cov_3.71969	32.97%	18899	29	Arctic Ocean	5	50.84	AAI-based (high-confidence)	OM43_clade	0.179
II	Station201_DCM_ALL_assembly_NODE_2223_length_23666_cov_9.88916	33.35%	23666	36	Arctic Ocean	36	62	AAI-based (high-confidence)	OM43_clade	0.225
II	Station201_SUR_ALL_assembly_NODE_2148_length_24684_cov_6.57188	32.89%	24684	39	Arctic Ocean	5	62.44	AAI-based (high-confidence)	OM43_clade	0.225
II	Station206_MES_ALL_assembly_NODE_2859_length_27141_cov_15.4362	33.19%	27141	42	Arctic Ocean	411	68.65	AAI-based (high-confidence)	OM43_clade	0.225
II	Station208_SUR_ALL_assembly_NODE_1344_length_27476_cov_10.2722	33.19%	27476	44	Arctic Ocean	5	69.46	AAI-based (high-confidence)	OM43_clade	0.228
II	IMGVR_UViG_3300001450_000204|3300001450|JGI24006J15134_100003	32.94%	27650	42	Subarctic Pacific Ocean	10	69.33	AAI-based (high-confidence)	OM43_clade	0.231
II	IMGVR_UViG_3300025168_000229|3300025168|Ga0209337_1000319	33.51%	37720	52	Subarctic Pacific Ocean	10	100	AAI-based (high-confidence)	OM43_clade	0.2
II	Station209_SUR_ALL_assembly_NODE_3251_length_23277_cov_117.626	34.58%	23277	36	Arctic Ocean	5	62.45	AAI-based (high-confidence)	Methylophilaceae	NA
II	Station82_SUR_COMBINED_FINAL_NODE_1640_length_21266_cov_4.234360	33.64%	21266	31	South Atlantic Ocean	5	57.52	AAI-based (high-confidence)	NA	NA
II	Station194_DCM_ALL_assembly_NODE_1131_length_35573_cov_8.73053	32.45%	35573	55	Arctic Ocean	35	100	AAI-based (high-confidence)	OM43_clade	0.166
II	DTR_891301	32.44%	35518	55	NA		100	AAI-based (high-confidence)	OM43_clade	0.166
II	Station173_DCM_ALL_assembly_NODE_2308_length_35401_cov_5.84510	34.57%	35401	53	Arctic Ocean	35	98.36	AAI-based (high-confidence)	OM43_clade	0.192
II	IMGVR_UViG_3300027668_000056|3300027668|Ga0209482_1000527	34.16%	32646	48	West Antarctic Peninsula	1	90.7	AAI-based (high-confidence)	OM43_clade	0.212
II	Station188_SUR_ALL_assembly_NODE_1877_length_30316_cov_138.806	34.97%	30316	43	Arctic Ocean	5	84.79	AAI-based (high-confidence)	OM43_clade	0.189
II	Station158_DCM_ALL_assembly_NODE_1718_length_36818_cov_7.16396	35.49%	36818	53	Arctic Ocean	25	99.47	AAI-based (high-confidence)	OM43_clade	NA
II	IMGVR_UViG_3300009512_000084|3300009512|Ga0115003_10001199	36.10%	23006	32	western Arctic Ocean	25	58.12	AAI-based (high-confidence)	OM43_clade	0.243

II	IMGVR_UViG_3300001450_000447|3300001450|JGI24006J15134_100007	32.28%	20173	34	Pacific Ocean	10	56.18	AAI-based (high-confidence)	NA	NA
II	IMGVR_UViG_3300025168_000832|3300025168|Ga0209337_1001091	32.30%	20968	35	Subarctic Pacific Ocean	10	58.39	AAI-based (high-confidence)	OM43_clade	0.261
II	IMGVR_UViG_3300000115_000230|3300000115|DelMOSum2011_c10000	32.52%	22281	36	Delaware Coast	0	59.7	AAI-based (high-confidence)	OM43_clade	0.244
II	IMGVR_UViG_3300000101_000203|3300000101|DelMOSum2010_c10001	32.51%	22280	36	Delaware Coast	0	59.7	AAI-based (high-confidence)	OM43_clade	0.242
II	IMGVR_UViG_3300007345_000256|3300007345|Ga0070752_1000752	33.29%	24934	45	USA: Delaware Bay	NA	62.66	AAI-based (medium-confidence)	OM43_clade	0.173
II	IMGVR_UViG_3300022187_000223|3300022187|Ga0196899_1000350	33.38%	24916	44	USA: Delaware Bay	NA	62.67	AAI-based (medium-confidence)	OM43_clade	0.179
II	IMGVR_UViG_3300025853_000568|3300025853|Ga0208645_1001054	33.40%	25724	46	USA: Delaware Bay	NA	64.7	AAI-based (medium-confidence)	OM43_clade	0.179
II	IMGVR_UViG_3300006810_000458|3300006810|Ga0070754_10001132	33.54%	21969	36	USA: Delaware Bay	NA	55.32	AAI-based (medium-confidence)	OM43_clade	0.176
II	IMGVR_UViG_3300025671_000231|3300025671|Ga0208898_1000378	33.83%	35061	57	USA: Delaware Bay	NA	96.05	AAI-based (high-confidence)	OM43_clade	0.16
II	IMGVR_UViG_3300007542_000201|3300007542|Ga0099846_1000230	38.54%	43449	30	USA: Chesapeake Bay	1	57.72	AAI-based (medium-confidence)	Xanthomona	0.352
II	IMGVR_UViG_3300006802_000966|3300006802|Ga0070749_10001118	34.03%	18138	30	USA: Delaware Bay	NA	50.03	AAI-based (high-confidence)	Vibrio	0.156
II	IMGVR_UViG_3300018048_000066|3300018048|Ga0181606_10001126	35.35%	23211	47	Groves Creek Marsh, Skidaway Island,	0	62.68	AAI-based (high-confidence)	NA	NA
II	IMGVR_UViG_3300025889_000319|3300025889|Ga0208644_1000301	35.14%	38916	58	USA: Delaware Bay	NA	100	AAI-based (high-confidence)	OM43_clade	NA
II	Station206_SUR_ALL_assembly_NODE_1246_length_34088_cov_7.50885	37.81%	34088	55	Arctic Ocean	5	93.12	AAI-based (high-confidence)	Roseobacter	0.147
II	Station205_SUR_ALL_assembly_NODE_886_length_33890_cov_838.4409	40.45%	33890	54	Arctic Ocean	5	96.59	AAI-based (high-confidence)	Roseobacter	0.157
II	IMGVR_UViG_3300007346_000254|3300007346|Ga0070753_1000519	33.63%	23655	40	USA: Delaware Bay	NA	63.61	AAI-based (high-confidence)	Pseudomona	0.15

II	IMGVR_UViG_3300007344_000296|3300007344|Ga0070745_1000574	34.00%	21923	40	USA: Delaware Bay	NA	56.52	AAI-based (high-confidence)	Pseudomonas	0.151
II	Station180_SUR_ALL_assembly_NODE_4506_length_23816_cov_3.59054	36.62%	23816	36	Arctic Ocean	5	64.04	AAI-based (high-confidence)	Roseobacter	0.151

Supplementary Table 3. Summary of linear regression analyses of the relative abundance of each MEP401-type phage with environmental parameters.

Depth (m)			Temperature(℃)			Salinity(psu)			Chlorophyll a, areal concentration (Chlora areal; mg/m2)		
**MEP401-type phages**	**p-value**	**R^2**	**MEP401-type phages**	**p-value**	**R^2**	**MEP401-type phages**	**p-value**	**R^2**	**MEP401-type phages**	**p-value**	**R^2**
DelMOSpr2010_c10000288	NA	NA	DelMOSpr2010_c10000288	NA	NA	DelMOSpr2010_c10000288	NA	NA	DelMOSpr2010_c10000288	NA	NA
DelMOSum2010_c10001088	0.3063	0.0081	DelMOSum2010_c10001088	0.0308	0.0362	DelMOSum2010_c10001088	0.0000	0.1537	DelMOSum2010_c10001088	0.3008	0.0085
Ga0114933_10000499	NA	NA	Ga0114933_10000499	NA	NA	Ga0114933_10000499	NA	NA	Ga0114933_10000499	NA	NA
Ga0114934_10000099	NA	NA	Ga0114934_10000099	NA	NA	Ga0114934_10000099	NA	NA	Ga0114934_10000099	NA	NA
Ga0181420_1000024	0.4048	0.0054	Ga0181420_1000024	0.0095	0.0518	Ga0181420_1000024	0.0943	0.0219	Ga0181420_1000024	0.4593	0.0044
Ga0181430_1000035	0.4021	0.0054	Ga0181430_1000035	0.0088	0.0529	Ga0181430_1000035	0.0916	0.0222	Ga0181430_1000035	0.4558	0.0044
Ga0181595_10000739	0.1905	0.0133	Ga0181595_10000739	0.0000	0.1662	Ga0181595_10000739	0.0000	0.3255	Ga0181595_10000739	0.5861	0.0024
Ga0181596_10000902	0.1554	0.0156	Ga0181596_10000902	0.0000	0.1879	Ga0181596_10000902	0.0000	0.3255	Ga0181596_10000902	0.9565	0.0000
Ga0181600_10000418	0.1713	0.0145	Ga0181600_10000418	0.0000	0.1783	Ga0181600_10000418	0.0000	0.3266	Ga0181600_10000418	0.5283	0.0032
Ga0181601_10000463	0.1715	0.0144	Ga0181601_10000463	0.0000	0.1784	Ga0181601_10000463	0.0000	0.3266	Ga0181601_10000463	0.5290	0.0032
Ga0181604_10000662	0.1511	0.0159	Ga0181604_10000662	0.0000	0.1907	Ga0181604_10000662	0.0000	0.3333	Ga0181604_10000662	0.9377	0.0000
Ga0181606_10000740	NA	NA	Ga0181606_10000740	NA	NA	Ga0181606_10000740	NA	NA	Ga0181606_10000740	NA	NA
Ga0196899_1000398	NA	NA	Ga0196899_1000398	NA	NA	Ga0196899_1000398	NA	NA	Ga0196899_1000398	NA	NA
Ga0208644_1000566	NA	NA	Ga0208644_1000566	NA	NA	Ga0208644_1000566	NA	NA	Ga0208644_1000566	NA	NA

Ga0208644_1000578	NA	NA	Ga0208644_1000578	NA	NA	Ga0208644_1000578	NA	NA	Ga0208644_1000578	NA	NA
Ga0208645_1001202	NA	NA	Ga0208645_1001202	NA	NA	Ga0208645_1001202	NA	NA	Ga0208645_1001202	NA	NA
Ga0208767_1000435	NA	NA	Ga0208767_1000435	NA	NA	Ga0208767_1000435	NA	NA	Ga0208767_1000435	NA	NA
Ga0208898_1000330	NA	NA	Ga0208898_1000330	NA	NA	Ga0208898_1000330	NA	NA	Ga0208898_1000330	NA	NA
Ga0208899_1000774	NA	NA	Ga0208899_1000774	NA	NA	Ga0208899_1000774	NA	NA	Ga0208899_1000774	NA	NA
Ga0209119_1001486	0.2139	0.0120	Ga0209119_1001486	0.0000	0.1513	Ga0209119_1001486	0.0000	0.3294	Ga0209119_1001486	0.2849	0.0091
Ga0209336_10000081	0.1903	0.0133	Ga0209336_10000081	0.0000	0.2786	Ga0209336_10000081	0.0000	0.1967	Ga0209336_10000081	0.2602	0.0100
Ga0209337_1000466	0.5237	0.0032	Ga0209337_1000466	0.0001	0.1171	Ga0209337_1000466	0.0028	0.0679	Ga0209337_1000466	0.0465	0.0311
Ga0209337_1000737	0.1895	0.0133	Ga0209337_1000737	0.0000	0.2799	Ga0209337_1000737	0.0000	0.1873	Ga0209337_1000737	0.2346	0.0112
Ga0209425_10001150	0.1704	0.0145	Ga0209425_10001150	0.0000	0.1834	Ga0209425_10001150	0.0000	0.3320	Ga0209425_10001150	0.5152	0.0034
Ga0209535_1000182	0.1900	0.0133	Ga0209535_1000182	0.0000	0.2791	Ga0209535_1000182	0.0000	0.1965	Ga0209535_1000182	0.2562	0.0102
Ga0209816_1000437	0.0675	0.0257	Ga0209816_1000437	0.0000	0.2723	Ga0209816_1000437	0.0000	0.2192	Ga0209816_1000437	0.1355	0.0176
Ga0222713_10000513	NA	NA	Ga0222713_10000513	NA	NA	Ga0222713_10000513	NA	NA	Ga0222713_10000513	NA	NA
Ga0222713_10001066	NA	NA	Ga0222713_10001066	NA	NA	Ga0222713_10001066	NA	NA	Ga0222713_10001066	NA	NA
Ga0222714_10001281	NA	NA	Ga0222714_10001281	NA	NA	Ga0222714_10001281	NA	NA	Ga0222714_10001281	NA	NA
Ga0222715_10000395	NA	NA	Ga0222715_10000395	NA	NA	Ga0222715_10000395	NA	NA	Ga0222715_10000395	NA	NA
Ga0222719_10001277	NA	NA	Ga0222719_10001277	NA	NA	Ga0222719_10001277	NA	NA	Ga0222719_10001277	NA	NA
Ga0233427_10001280	0.1903	0.0133	Ga0233427_10001280	0.0000	0.2873	Ga0233427_10001280	0.0000	0.1833	Ga0233427_10001280	0.2108	0.0124

Ga0233433_10000462	0.2068	0.0123	Ga0233433_10000462	0.0000	0.2726	Ga0233433_10000462	0.0000	0.1836	Ga0233433_10000462	0.2398	0.0110
Ga0233434_1000496	0.2067	0.0123	Ga0233434_1000496	0.0000	0.2728	Ga0233434_1000496	0.0000	0.1836	Ga0233434_1000496	0.2390	0.0110
Ga0233435_1000531	0.2068	0.0123	Ga0233435_1000531	0.0000	0.2728	Ga0233435_1000531	0.0000	0.1837	Ga0233435_1000531	0.2391	0.0110
Ga0233436_1000489	0.2082	0.0123	Ga0233436_1000489	0.0000	0.2721	Ga0233436_1000489	0.0000	0.1832	Ga0233436_1000489	0.2410	0.0109
Ga0233439_10000765	0.2065	0.0123	Ga0233439_10000765	0.0000	0.2719	Ga0233439_10000765	0.0000	0.1842	Ga0233439_10000765	0.2461	0.0107
Ga0233439_10001445	0.9416	0.0000	Ga0233439_10001445	0.0000	0.1822	Ga0233439_10001445	0.0110	0.0498	Ga0233439_10001445	0.0169	0.0444
Ga0233440_1000494	0.2062	0.0124	Ga0233440_1000494	0.0000	0.2738	Ga0233440_1000494	0.0000	0.1873	Ga0233440_1000494	0.2440	0.0108
Ga0233442_1000411	0.2068	0.0123	Ga0233442_1000411	0.0000	0.2728	Ga0233442_1000411	0.0000	0.1836	Ga0233442_1000411	0.2390	0.0110
Ga0233443_1000467	0.2083	0.0122	Ga0233443_1000467	0.0000	0.2722	Ga0233443_1000467	0.0000	0.1834	Ga0233443_1000467	0.2410	0.0109
Ga0233445_1000475	0.2072	0.0123	Ga0233445_1000475	0.0000	0.2729	Ga0233445_1000475	0.0000	0.1868	Ga0233445_1000475	0.2461	0.0107
Ga0233446_1000450	0.2082	0.0123	Ga0233446_1000450	0.0000	0.2720	Ga0233446_1000450	0.0000	0.1831	Ga0233446_1000450	0.2410	0.0109
Ga0233447_1000483	0.2094	0.0122	Ga0233447_1000483	0.0000	0.2716	Ga0233447_1000483	0.0000	0.1831	Ga0233447_1000483	0.2420	0.0108
Ga0233448_1000461	0.2084	0.0122	Ga0233448_1000461	0.0000	0.2720	Ga0233448_1000461	0.0000	0.1830	Ga0233448_1000461	0.2406	0.0109
Ga0233449_1000537	0.2083	0.0122	Ga0233449_1000537	0.0000	0.2720	Ga0233449_1000537	0.0000	0.1832	Ga0233449_1000537	0.2415	0.0109
Ga0255753_1001477	NA	NA	Ga0255753_1001477	NA	NA	Ga0255753_1001477	NA	NA	Ga0255753_1001477	NA	NA
Ga0255765_1001150	0.1703	0.0145	Ga0255765_1001150	0.0000	0.1785	Ga0255765_1001150	0.0000	0.3300	Ga0255765_1001150	0.5199	0.0033
Ga0308009_10000676	0.9328	0.0001	Ga0308009_10000676	0.0000	0.2070	Ga0308009_10000676	0.0023	0.0710	Ga0308009_10000676	0.0073	0.0558
Ga0308015_10000292	0.9092	0.0001	Ga0308015_10000292	0.0000	0.1989	Ga0308015_10000292	0.0036	0.0647	Ga0308015_10000292	0.0133	0.0476

JGI20154J14316_10000991	0.2058	0.0124	JGI20154J14316_10000991	0.0000	0.1510	JGI20154J14316_10000991	0.0000	0.3307	JGI20154J14316_10000991	0.2806	0.0092
JGI20156J14371_10000598	0.1721	0.0144	JGI20156J14371_10000598	0.0000	0.1818	JGI20156J14371_10000598	0.0000	0.3265	JGI20156J14371_10000598	0.5263	0.0032
MEP401	0.1892	0.0133	MEP401	0.0000	0.2760	MEP401	0.0000	0.2058	MEP401	0.3452	0.0071
MEP402	0.2016	0.0126	MEP402	0.0000	0.2585	MEP402	0.0000	0.1750	MEP402	0.2370	0.0111
Station102_SUR_ALL_assembly_NODE_628_length_25877_cov_2.854117	0.4615	0.0042	Station102_SUR_ALL_assembly_NODE_628_length_25877_cov_2.854117	0.0033	0.0658	Station102_SUR_ALL_assembly_NODE_628_length_25877_cov_2.854117	0.0047	0.0611	Station102_SUR_ALL_assembly_NODE_628_length_25877_cov_2.854117	0.6248	0.0019
Station168_DCM_ALL_assembly_NODE_1952_length_39344_cov_140.040469	0.1679	0.0147	Station168_DCM_ALL_assembly_NODE_1952_length_39344_cov_140.040469	0.0000	0.3214	Station168_DCM_ALL_assembly_NODE_1952_length_39344_cov_140.040469	0.0006	0.0890	Station168_DCM_ALL_assembly_NODE_1952_length_39344_cov_140.040469	0.0014	0.0780
Station168_IZZ_ALL_assembly_NODE_3354_length_27763_cov_214.623719	0.1661	0.0148	Station168_IZZ_ALL_assembly_NODE_3354_length_27763_cov_214.623719	0.0000	0.3144	Station168_IZZ_ALL_assembly_NODE_3354_length_27763_cov_214.623719	0.0008	0.0843	Station168_IZZ_ALL_assembly_NODE_3354_length_27763_cov_214.623719	0.0014	0.0778
Station180_ZZZ_ALL_assembly_NODE_2619_length_24406_cov_10.970720	0.6426	0.0017	Station180_ZZZ_ALL_assembly_NODE_2619_length_24406_cov_10.970720	0.0000	0.1809	Station180_ZZZ_ALL_assembly_NODE_2619_length_24406_cov_10.970720	0.0059	0.0582	Station180_ZZZ_ALL_assembly_NODE_2619_length_24406_cov_10.970720	0.0318	0.0361
Station188_DCM_ALL_assembly_NODE_1692_length_30884_cov_155.533199	0.1569	0.0155	Station188_DCM_ALL_assembly_NODE_1692_length_30884_cov_155.533199	0.0000	0.3014	Station188_DCM_ALL_assembly_NODE_1692_length_30884_cov_155.533199	0.0017	0.0752	Station188_DCM_ALL_assembly_NODE_1692_length_30884_cov_155.533199	0.0015	0.0772
Station188_SUR_ALL_assembly_NODE_2046_length_28441_cov_114.578384	0.1590	0.0153	Station188_SUR_ALL_assembly_NODE_2046_length_28441_cov_114.578384	0.0000	0.3084	Station188_SUR_ALL_assembly_NODE_2046_length_28441_cov_114.578384	0.0011	0.0811	Station188_SUR_ALL_assembly_NODE_2046_length_28441_cov_114.578384	0.0018	0.0744
Station193_SUR_ALL_assembly_NODE_727_length_39660_cov_39.387855	0.0793	0.0237	Station193_SUR_ALL_assembly_NODE_727_length_39660_cov_39.387855	0.0000	0.2409	Station193_SUR_ALL_assembly_NODE_727_length_39660_cov_39.387855	0.0000	0.2637	Station193_SUR_ALL_assembly_NODE_727_length_39660_cov_39.387855	0.2887	0.0089
Station194_SUR_ALL_assembly_NODE_975_length_31631_cov_35.550576	0.1279	0.0179	Station194_SUR_ALL_assembly_NODE_975_length_31631_cov_35.550576	0.0000	0.3622	Station194_SUR_ALL_assembly_NODE_975_length_31631_cov_35.550576	0.0000	0.1420	Station194_SUR_ALL_assembly_NODE_975_length_31631_cov_35.550576	0.0051	0.0605
Station210_MES_ALL_assembly_NODE_1866_length_31658_cov_9.707623	0.1633	0.0150	Station210_MES_ALL_assembly_NODE_1866_length_31658_cov_9.707623	0.0000	0.3188	Station210_MES_ALL_assembly_NODE_1866_length_31658_cov_9.707623	0.0007	0.0875	Station210_MES_ALL_assembly_NODE_1866_length_31658_cov_9.707623	0.0015	0.0773

Station23_DCM_ALL_assembly_NODE_744_length_20427_cov_3.425093	0.1748	0.0142	Station23_DCM_ALL_assembly_NODE_744_length_20427_cov_3.425093	0.0000	0.2973	Station23_DCM_ALL_assembly_NODE_744_length_20427_cov_3.425093	0.0005	0.0911	Station23_DCM_ALL_assembly_NODE_744_length_20427_cov_3.425093	0.0102	0.0512
DelMOSum2010_c10001091	0.2304	0.0111	DelMOSum2010_c10001091	0.0000	0.1396	DelMOSum2010_c10001091	0.0300	0.0365	DelMOSum2010_c10001091	0.1161	0.0195
DelMOSum2011_c10000730	0.2308	0.0111	DelMOSum2011_c10000730	0.0000	0.1396	DelMOSum2011_c10000730	0.0297	0.0367	DelMOSum2011_c10000730	0.1171	0.0194
DTR_891301	0.8777	0.0002	DTR_891301	0.0007	0.0867	DTR_891301	0.0004	0.0956	DTR_891301	0.9323	0.0001
Ga0070745_1000574	NA	NA	Ga0070745_1000574	NA	NA	Ga0070745_1000574	NA	NA	Ga0070745_1000574	NA	NA
Ga0070749_10001118	NA	NA	Ga0070749_10001118	NA	NA	Ga0070749_10001118	NA	NA	Ga0070749_10001118	NA	NA
Ga0070752_1000752	NA	NA	Ga0070752_1000752	NA	NA	Ga0070752_1000752	NA	NA	Ga0070752_1000752	NA	NA
Ga0070753_1000519	NA	NA	Ga0070753_1000519	NA	NA	Ga0070753_1000519	NA	NA	Ga0070753_1000519	NA	NA
Ga0070754_10001132	NA	NA	Ga0070754_10001132	NA	NA	Ga0070754_10001132	NA	NA	Ga0070754_10001132	NA	NA
Ga0099846_1000230	NA	NA	Ga0099846_1000230	NA	NA	Ga0099846_1000230	NA	NA	Ga0099846_1000230	NA	NA
Ga0115003_10001199	0.4075	0.0053	Ga0115003_10001199	0.0000	0.1480	Ga0115003_10001199	0.0004	0.0946	Ga0115003_10001199	0.3289	0.0076
Ga0181606_10001126	NA	NA	Ga0181606_10001126	NA	NA	Ga0181606_10001126	NA	NA	Ga0181606_10001126	NA	NA
Ga0196899_1000350	NA	NA	Ga0196899_1000350	NA	NA	Ga0196899_1000350	NA	NA	Ga0196899_1000350	NA	NA
Ga0208644_1000301	NA	NA	Ga0208644_1000301	NA	NA	Ga0208644_1000301	NA	NA	Ga0208644_1000301	NA	NA
Ga0208645_1001054	NA	NA	Ga0208645_1001054	NA	NA	Ga0208645_1001054	NA	NA	Ga0208645_1001054	NA	NA
Ga0208898_1000378	NA	NA	Ga0208898_1000378	NA	NA	Ga0208898_1000378	NA	NA	Ga0208898_1000378	NA	NA
Ga0209337_1000319	0.6467	0.0016	Ga0209337_1000319	0.0000	0.1884	Ga0209337_1000319	0.0002	0.1030	Ga0209337_1000319	0.1596	0.0156
Ga0209337_1001091	0.5461	0.0028	Ga0209337_1001091	0.9129	0.0001	Ga0209337_1001091	0.6582	0.0015	Ga0209337_1001091	0.2725	0.0096

Ga0209482_1000527	0.0788	0.0238	Ga0209482_1000527	0.0000	0.2123	Ga0209482_1000527	0.0000	0.4694	Ga0209482_1000527	0.7973	0.0005
JGI24006J15134_10000376	0.7137	0.0010	JGI24006J15134_10000376	0.0000	0.1519	JGI24006J15134_10000376	0.0004	0.0955	JGI24006J15134_10000376	0.5148	0.0034
JGI24006J15134_10000703	0.6951	0.0012	JGI24006J15134_10000703	0.9796	0.0000	JGI24006J15134_10000703	0.6606	0.0015	JGI24006J15134_10000703	0.3284	0.0076
Station158_DCM_ALL_assembly_NODE_1718_length_36818_cov_7.163969	0.3935	0.0057	Station158_DCM_ALL_assembly_NODE_1718_length_36818_cov_7.163969	0.0001	0.1105	Station158_DCM_ALL_assembly_NODE_1718_length_36818_cov_7.163969	0.0008	0.0845	Station158_DCM_ALL_assembly_NODE_1718_length_36818_cov_7.163969	0.5161	0.0034
Station173_DCM_ALL_assembly_NODE_2308_length_35401_cov_5.845103	0.0792	0.0237	Station173_DCM_ALL_assembly_NODE_2308_length_35401_cov_5.845103	0.0000	0.2257	Station173_DCM_ALL_assembly_NODE_2308_length_35401_cov_5.845103	0.0000	0.4552	Station173_DCM_ALL_assembly_NODE_2308_length_35401_cov_5.845103	0.9145	0.0001
Station180_SUR_ALL_assembly_NODE_4506_length_23816_cov_3.590548	0.4657	0.0041	Station180_SUR_ALL_assembly_NODE_4506_length_23816_cov_3.590548	0.1113	0.0198	Station180_SUR_ALL_assembly_NODE_4506_length_23816_cov_3.590548	0.0000	0.2474	Station180_SUR_ALL_assembly_NODE_4506_length_23816_cov_3.590548	0.3937	0.0058
Station188_SUR_ALL_assembly_NODE_1877_length_30316_cov_138.806451	0.1544	0.0157	Station188_SUR_ALL_assembly_NODE_1877_length_30316_cov_138.806451	0.0000	0.3152	Station188_SUR_ALL_assembly_NODE_1877_length_30316_cov_138.806451	0.0000	0.1410	Station188_SUR_ALL_assembly_NODE_1877_length_30316_cov_138.806451	0.1133	0.0198
Station194_DCM_ALL_assembly_NODE_1131_length_35573_cov_8.730531	0.8784	0.0002	Station194_DCM_ALL_assembly_NODE_1131_length_35573_cov_8.730531	0.0007	0.0868	Station194_DCM_ALL_assembly_NODE_1131_length_35573_cov_8.730531	0.0004	0.0956	Station194_DCM_ALL_assembly_NODE_1131_length_35573_cov_8.730531	0.9322	0.0001
Station201_DCM_ALL_assembly_NODE_2223_length_23666_cov_9.889162	0.6103	0.0020	Station201_DCM_ALL_assembly_NODE_2223_length_23666_cov_9.889162	0.0000	0.1939	Station201_DCM_ALL_assembly_NODE_2223_length_23666_cov_9.889162	0.0003	0.0994	Station201_DCM_ALL_assembly_NODE_2223_length_23666_cov_9.889162	0.1047	0.0208
Station201_SUR_ALL_assembly_NODE_2148_length_24684_cov_6.571887	0.6425	0.0017	Station201_SUR_ALL_assembly_NODE_2148_length_24684_cov_6.571887	0.0000	0.1579	Station201_SUR_ALL_assembly_NODE_2148_length_24684_cov_6.571887	0.0007	0.0877	Station201_SUR_ALL_assembly_NODE_2148_length_24684_cov_6.571887	0.2723	0.0096
Station205_SUR_ALL_assembly_NODE_886_length_33890_cov_838.440934	0.4778	0.0039	Station205_SUR_ALL_assembly_NODE_886_length_33890_cov_838.440934	0.0225	0.0403	Station205_SUR_ALL_assembly_NODE_886_length_33890_cov_838.440934	0.0247	0.0391	Station205_SUR_ALL_assembly_NODE_886_length_33890_cov_838.440934	0.6824	0.0013

Station206_MES_ALL_assembly_NODE_2859_length_27141_cov_15.436240	0.5630	0.0026	Station206_MES_ALL_assembly_NODE_2859_length_27141_cov_15.436240	0.0000	0.1794	Station206_MES_ALL_assembly_NODE_2859_length_27141_cov_15.436240	0.0006	0.0878	Station206_MES_ALL_assembly_NODE_2859_length_27141_cov_15.436240	0.0433	0.0320
Station206_SUR_ALL_assembly_NODE_1246_length_34088_cov_7.508859	0.8843	0.0002	Station206_SUR_ALL_assembly_NODE_1246_length_34088_cov_7.508859	0.0176	0.0436	Station206_SUR_ALL_assembly_NODE_1246_length_34088_cov_7.508859	0.0392	0.0330	Station206_SUR_ALL_assembly_NODE_1246_length_34088_cov_7.508859	0.9841	3.17E-06
Station206_SUR_ALL_assembly_NODE_3088_length_18899_cov_3.719699	0.7185	0.0010	Station206_SUR_ALL_assembly_NODE_3088_length_18899_cov_3.719699	0.0000	0.1432	Station206_SUR_ALL_assembly_NODE_3088_length_18899_cov_3.719699	0.0008	0.0843	Station206_SUR_ALL_assembly_NODE_3088_length_18899_cov_3.719699	0.5147	0.0034
Station208_SUR_ALL_assembly_NODE_1344_length_27476_cov_10.272273	0.5996	0.0021	Station208_SUR_ALL_assembly_NODE_1344_length_27476_cov_10.272273	0.0000	0.1929	Station208_SUR_ALL_assembly_NODE_1344_length_27476_cov_10.272273	0.0002	0.1034	Station208_SUR_ALL_assembly_NODE_1344_length_27476_cov_10.272273	0.0778	0.0245
Station209_SUR_ALL_assembly_NODE_3251_length_23277_cov_117.626647	0.1503	0.0160	Station209_SUR_ALL_assembly_NODE_3251_length_23277_cov_117.626647	0.0000	0.3439	Station209_SUR_ALL_assembly_NODE_3251_length_23277_cov_117.626647	0.0001	0.1129	Station209_SUR_ALL_assembly_NODE_3251_length_23277_cov_117.626647	0.0042	0.0631
Station209_SUR_ALL_assembly_NODE_3481_length_22302_cov_43.575448	0.6905	0.0012	Station209_SUR_ALL_assembly_NODE_3481_length_22302_cov_43.575448	0.0000	0.1508	Station209_SUR_ALL_assembly_NODE_3481_length_22302_cov_43.575448	0.0004	0.0944	Station209_SUR_ALL_assembly_NODE_3481_length_22302_cov_43.575448	0.4975	0.0037
Station82_SUR_COMBINED_FINAL_NODE_1640_length_21266_cov_4.234360	0.6289	0.0018	Station82_SUR_COMBINED_FINAL_NODE_1640_length_21266_cov_4.234360	0.0000	0.1770	Station82_SUR_COMBINED_FINAL_NODE_1640_length_21266_cov_4.234360	0.0004	0.0954	Station82_SUR_COMBINED_FINAL_NODE_1640_length_21266_cov_4.234360	0.1388	0.0173

Table S4 qPCR primer sequences used in this study

Primer set	Primer Sequence (5 -3)	GC(%)	Product size (bp)
MEP401-F/R	CAAGCTATGCATGGTTGGGT	50%	132
	CCTACAAGCACATCGCACTT	50%	
MEP402-F/R	GCATTGGCTACGCAGGTATG	55%	104
	AGGATGGGTAAGCTAGGTCTTG	50%	
